# A critical review of the impacts of cover crops on nitrogen leaching, net greenhouse gas balance and crop productivity

**DOI:** 10.1111/gcb.14644

**Published:** 2019-05-13

**Authors:** Mohamed Abdalla, Astley Hastings, Kun Cheng, Qian Yue, Dave Chadwick, Mikk Espenberg, Jaak Truu, Robert M. Rees, Pete Smith

**Affiliations:** ^1^ Institute of Biological and Environmental Sciences, School of Biological Sciences University of Aberdeen Aberdeen UK; ^2^ Institute of Resource, Ecosystem and Environment of Agriculture, Centre of Climate Change and Agriculture Nanjing Agricultural University Nanjing Jiangsu China; ^3^ School of Natural Resources Bangor University Bangor Gwynedd UK; ^4^ Faculty of Science and Technology University of Tartu Tartu Estonia; ^5^ Scotland's Rural College (SRUC) Edinburgh Edinburgh UK

**Keywords:** C sequestration, catch crop, cover crop, green manure, N content, N in grain, N leaching, net greenhouse gas balance, nitrate, nitrous oxide emissions, soil organic carbon, yield

## Abstract

Cover crops play an increasingly important role in improving soil quality, reducing agricultural inputs and improving environmental sustainability. The main objectives of this critical global review and systematic analysis were to assess cover crop practices in the context of their impacts on nitrogen leaching, net greenhouse gas balances (NGHGB) and crop productivity. Only studies that investigated the impacts of cover crops and measured one or a combination of nitrogen leaching, soil organic carbon (SOC), nitrous oxide (N_2_O), grain yield and nitrogen in grain of primary crop, and had a control treatment were included in the analysis. Long‐term studies were uncommon, with most data coming from studies lasting 2–3 years. The literature search resulted in 106 studies carried out at 372 sites and covering different countries, climatic zones and management. Our analysis demonstrates that cover crops significantly (*p* < 0.001) decreased N leaching and significantly (*p* < 0.001) increased SOC sequestration without having significant (*p* > 0.05) effects on direct N_2_O emissions. Cover crops could mitigate the NGHGB by 2.06 ± 2.10 Mg CO_2_‐eq ha^−1^ year^−1^. One of the potential disadvantages of cover crops identified was the reduction in grain yield of the primary crop by ≈4%, compared to the control treatment. This drawback could be avoided by selecting mixed cover crops with a range of legumes and non‐legumes, which increased the yield by ≈13%. These advantages of cover crops justify their widespread adoption. However, management practices in relation to cover crops will need to be adapted to specific soil, management and regional climatic conditions.

## Graphical abstract

Cover crops significantly (*p* < 0.001) decreased N leaching and significantly (*p* < 0.001) increased soil organic carbon sequestration without having significant (*p* > 0.05) effects on direct N_2_O emissions. Cover crops could mitigate net greenhouse gas balance by 2.06 ± 2.10 Mg CO_2_‐eq ha^−1^ year^−1^. One of the potential disadvantages of the cover crops identified was the reduction in grain yield of the primary crop by ≈4%, compared to the control treatment. This drawback could be avoided by selecting legume–non‐legume mixed cover crops. However, cover crop management need to be adapted to specific soil, management and regional climatic conditions.

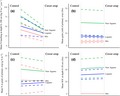

## 1 INTRODUCTION

Increasing crop productivity with reduced inputs and lower impacts on the environment is a major current challenge for global food production. Cover crops (also known as catch crops) are plants mostly grown after a primary crop is harvested, in regions of the world where only a single main crop is grown (such as North Europe, North China and Canada). This avoids periods of bare soil which are associated with greater risk of erosion and nitrogen leaching losses (Battany & Grismer, 2000). Cover cropping can comprise a single species or a mixture of species and can use annual, biennial or perennial vegetation. Cover crops can be killed (or ploughed‐in) in winter or spring, or grazed, and incorporated in soils by tillage to prevent competition with the primary crop, and to promote mineralization of organic N (Dabney et al., 2011). They can also be left on the soil surface over the fall and winter periods, until a primary crop in no‐till is planted, to provide weed control and N inputs (Halde, Gulden, & Entz, 2014).

Cover crops can increase water holding capacity, soil porosity, aggregate stability, the size of the microbial population and its activity and nutrient cycling (Drinkwater & Snapp, 2007; Harunaa & Nkongolo, 2015; Lotter, Seidel, & Liebhardt, 2003). There are four classes of cover crops: legumes (e.g. alfalfa, vetches and clover), non‐legumes (spinach, canola and flax), grasses (e.g. ryegrass and barley) and brassicas (e.g. radishes and turnips). The two main types of cover crops are legumes and non‐legumes. Legume cover crops have the ability to fix nitrogen (N) biologically and increase soil organic matter (SOM) content (Lüscher, Mueller‐Harvey, Soussana, Rees, & Peyraud, 2014). They can be used as a green manure to improve soil nutrition for the subsequent primary crop. The non‐legume cover crops can absorb excess nitrate from the soil, increase crop biomass, and improve soil quality (Finney, White, & Kaye, 2016; White, Finney, Kemanian, & Kaye, 2016). Farmers, generally, select specific types of cover crops based on their own needs and goals influenced by biological, environmental, social, cultural and economic factors of the farming systems in which they operate (Snapp et al., 2005). Additionally, cover crops have become of greater interest for their potential to provide additional ecosystem services in agricultural systems (e.g. to reduce erosion, improve water quality and enhance biodiversity). In Spain, Hontoria, Garcia‐Gonzalez, Quemada, Roldan, and Alguacil (2019) found that the use of barley as a winter cover crops is an appropriate choice to promote arbuscular mycorrhizal fungal populations and biological activity in https://www.sciencedirect.com/topics/earth-and-planetary-sciences/agricultural-soil with intercropping systems.

Nitrogen leaching from agricultural soils is of great concern due to its contribution to excess nitrate (NO_3_) concentrations in ground water and run‐off (Ascott et al., 2017), indirect emissions of greenhouse gases (GHGs), for example, nitrous oxide (N_2_O) (Delgado et al., 2008), and loss of expensive N fertilizer (Cardenas et al., 2011). This problem is more pronounced in areas with fertilized coarse‐textured soils (Basche, Miguez, Kaspar, & Castellano, 2014) or areas with high precipitation (Thorup‐Kristensen, Magid, & Jensen, 2003). In England, Allingham et al. (2002) reported an average NO_3_ leaching value of 65 kg N/ha, which is approximately 25% of total N input. Similar NO_3_ losses, as a proportion of the total N applied, have been reported following livestock slurry and poultry manure applications to arable soils (Chambers, Smith, & Pain, 2000). Previous studies have found that replacing fallow periods with non‐legume cover crops is an effective management practice to withdraw soil N into the biomass of the cover crops and to reduce NO_3_ leaching (Basche et al., 2014; Kaspar & Singer, 2011; Quemada, Baranski, Nobel‐de Lange, Vallejo, & Cooper, 2013). Cover crops can also increase soil organic carbon (SOC) stocks in agricultural soils (Poeplau & Don, 2015), since more C and N are added to the soil pools as cover crop residues decompose (Kaspar & Singer, 2011; Steenwerth & Belina, 2008). The amounts of C and N incorporated into the soil depend on many factors, for example the amount, quality and management of the residues, soil type, frequency of tillage and climatic conditions (Smith et al., 2008; Stevenson, 1982). However, it is still not clear how cover crops affect the net greenhouse gas balance (NGHGB). Further, there is conflicting evidence on the influence of the cover crops on grain yields and N in the grain of primary crops. Some previous studies found that under‐sowing of cover crops in spring could lead to a high level of competition with the primary crop for nutrients, soil moisture and light, and result in some loss of the grain yield (Känkänen, Eriksson, Räkköläinen, & Vuorinen, 2001; Känkänen, Eriksson, Räkköläinen, & Vuorinrn, 2003; Karlsson‐Strese, Rydberg, Becker, & Umaerus, 1998). Other studies found that grain yield of the primary crops was not affected (Ohlander, Bergkvist, Stendahl, & Kvist, 1996; Wallgren & Lindén, 1994) or was even increased (Campiglia, Mancinelli, Radicetti, & Marinari, 2011). Mixed results have also been reported for the effects of cover crops on N in grain of the primary crop (Doltra & Olesen, 2013; Rinnofner, Friedel, Kruijff, Pietsch, & Freyer, 2008; Thomsen, 2014).

The main objectives of this global review and systematic analysis were to investigate the impacts of cover crops (legume, non‐legume and legume–non‐legume mixed) on N leaching, the NGHGB and crop productivity in terms of grain yield and N content in the grain of the primary crop. We also investigated whether soil characteristics, field management and climatic zones can modify these effects, and through this, we assessed the viability of cover crops as a management tool to enhance C sequestration, reduce N loss from agroecosystems and maintain crop production. The specific hypotheses we critically evaluated were as follows: (a) cover crops decrease N loss and increase SOC accumulation; (b) the impacts of cover crops on N loss and SOC are modified by soil, management and climatic zones; and (c) including cover crops in crop rotations improves grain yield and N in grain of the primary crop.

## 2 MATERIALS AND METHODS

### 2.1 Data collection

To analyse the publications that have investigated the impacts of cover crops on N leaching, SOC, N_2_O, grain yield and N in grain for different primary crops (e.g. wheat, barley, oats, corn and others), we made a comprehensive search on the Web of Science database (accessed between January 2017 and September 2018) using the keywords: Cover crop, Catch crop, N leaching, SOC, N in grain, nitrous oxide emissions, GHG balance, Green manure, Yield, N content, Nitrate and C sequestration. To gain the best possible coverage of the topic, we also checked all references in the papers collected from the Web of Science search. We only selected studies that investigated the effects of cover crops (legume, non‐legume and legume–non‐legume mixed), covered at least one growing season and measured one or a combination of: N leaching, SOC, N_2_O, grain yield and N in grain of primary crop, and had a control treatment. Nitrous oxide data were collected from studies that measured the gas flux from cropland and applied either a static or automated chamber method. SOC was measured as stocks (Mg/ha) but in some studies the values were given as concentrations. To convert these values to stocks, we applied Equation (1) below (Guo & Gifford, 2002):

(1) $$\text{Cs}\;=\;\left(\text{SOC}\;*\;\text{BD}\;*\;D\right)/10$$

where Cs is soil organic carbon stocks (Mg/ha), SOC is soil organic carbon concentration (g/kg), BD is bulk density (g/cm^3^) and *D* is soil depth (cm).

For SOC and N leaching data, we selected studies that measured them from zero and up to 30 and 100 cm soil depth respectively. To improve comparability of the different studies, we normalized the SOC data to the top 30 cm and the N leaching data to the top 100 cm depth, using the depth distribution method produced by Jobbágy and Jackson (2000) (Equations (2), (3), (4)).

(2) $$Y\;=\;1\;-\;\beta^{d}$$

(3) $${\text{SOC}}_{30}\;=\;\left(\left(1\;-\;\beta^{30}\right)/\left(1\;-\;\beta^{d0}\right)\right)\;*\;{\text{SOC}}_{d0}$$

(4) $$N_{100}\;=\;\left(\left(1\;-\;\beta^{100}\right)/\left(1\;-\;\beta^{d0}\right)\right)\;*\;N_{d0}$$

where *Y* is the cumulative proportion of the SOC or soil N leaching pool from the soil surface to depth *d* (cm) and *β* is the relative rate of decrease in the soil SOC or N pool with soil depth (0.9786 for SOC and 0.9831 for N) (Jobbágy & Jackson, 2001, 2000). SOC_30_ or N_100_ is the SOC (Mg/ha) or N (kg N/ha) pool in the upper 30 or 100 cm depth respectively; *d*_0_ is the original soil depth available in individual studies (cm); SOC*_d_*_0_ or N*_d_*_0_ is the original soil SOC or N pool.

We defined the control treatment as an annual fertilized primary crop with a bare fallow period between harvest and the establishment of the next primary crop. Where two main crops are grown synchronously, they are usually then referred to as intercrops, and such systems were not considered further in this review. We excluded many studies either because there was no control or because the experimental treatments did not meet the above criteria. Our literature search resulted in 106 studies carried out at 372 sites (Tables [Link], [Link], [Link], [Link], [Link]) that investigated the impacts of cover crops on N leaching, grain yield and N in grain of primary crop, SOC, N_2_O emissions, respectively, and covering different countries, climatic zones and management systems. The majority of the studies collected were short‐term experiments of 2–3 years. Locations, climatic conditions as well as primary crop, cover crops, type of cover crops (legume, non‐legume or legume–non‐legume mixed), study duration, tillage, N fertilizer application rate, soil texture, soil depth (cm), BD, soil pH and measurements from control and treatments, that is N leaching, grain yield, N in grain of primary crop, SOC and N_2_O, are shown in Tables [Link], [Link], [Link], [Link], [Link]. When there was more than 1 year of study in the original paper, we used the mean value for different years. We included different methods for measuring N leaching (e.g. field cores, ceramic suction cup lysimeter and subsurface drainage lysimeter). Nitrogen leaching was measured/calculated in kg N ha^−1^ year^−1^ whilst SOC and grain yield in t ha^−1^ year^−1^ and N in grain in g N m^−2^ year^−1^. We found 78% of the N leaching dataset collected had conventional tillage systems whilst the rest (22%) was divided between the different types of conservation tillage systems (i.e. no‐till, reduced till and minimum till) or had no data. Therefore, we investigated the influence of tillage on cover crop efficiency to reduce N leaching, N_2_O and SOC by comparing between conventional and conservation tillage systems.

To investigate the impacts of climate, we divided our dataset into four groups depending on the climatic zones. Climatic zones were distinguished on the basis of temperature and moisture regimes (cool, warm, dry and moist zone) to represent the global variations of soil moisture and temperature. The cool zone covers the temperate (oceanic, subcontinental and continental) and boreal (oceanic, subcontinental and continental) areas, whilst the warm zone covers the tropics (lowland and highland) and subtropical (summer rainfall, winter rainfall, and low rainfall) areas (Abdalla et al., 2018; Smith, Peters, Blackshaw, Lindwall, & Larney, 1996). The dry zone includes the areas where the annual precipitation is ≤500 mm, whilst the moist zone includes areas where the annual precipitation is >500 mm (Smith et al., 1996). The four climate categories were moist cool (MC), moist warm (MW), dry cool (DC) and dry warm (DW). However, to investigate the influences of climatic zones on the efficiency of cover crops to reduce N leaching and SOC, comparisons were made between the MC and MW only as most of the dataset belong to these two climatic zones: MC (68%) and MW (24%). The two other climatic zones both have only four observations.

**Figure 1:**
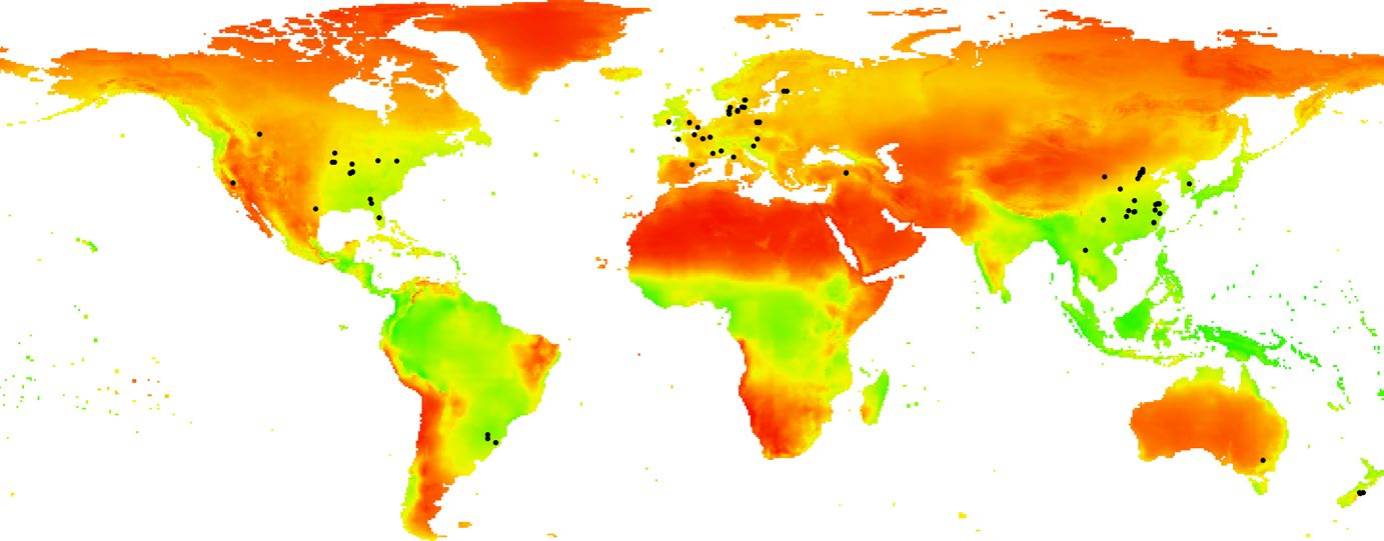
Map showing the net primary productivity (NPP) and locations of experimental sites considered in this paper. NPP calculated using the Miami method (Grieser et al., 2006; Leith, 1972)

For the different studies, different methods were used to measure soil pH, for example using a pH probe or meter in deionized water or 0.01 M CaCl_2_ in 1:1 and 1:2 or 1:5 (v:v) soils:solution ratios. We assumed the pH results to be equivalent, and where a range of values were reported, we took the arithmetic mean. Soil BD and pH from the different studies were measured from zero and up to 100 cm depth. The mean annual air temperature (MAAT, in °C) value and mean annual precipitation (MAP, in mm) values for each study were collected from the original published papers. The locations of experiments used in this study were plotted on a map of net primary production (NPP) calculated using the Miami method (Grieser, Gommes, & Bernardi, 2006; Leith, 1972), to indicate the diversity of arable capability included (Figure 1).

### 2.2 Direct/indirect N_2_O emissions and NGHGB

The direct N_2_O emissions data were collected from the literature (Table S5). Following Tier I IPCC protocol (IPCC, 2013) and Parkin, Kaspar, Jaynes, and Moorman (2016), we estimated the indirect N_2_O emissions for the control and cover crop treatments from the N leaching using the EF of 0.0075 multiplied by the mass of N leached. The change in the indirect N_2_O emissions due to cover crops was then calculated as shown in Table S1. The indirect emissions associated with NH_3_ and NO*x* were not estimated. The contributions of SOC (Table S4) and N_2_O to the NGHGB were calculated using the IPCC (2006) approach, where on a mass basis, N_2_O has a global warming potential (GWP) of 298 times that of CO_2_, over a 100‐year timescale. The methane (CH_4_) flux was considered to be negligible as, generally, cropland soils tend to be well drained and oxygenated and are often small net CH_4_ sinks (Abdalla et al., 2014; Lee, Six, King, van Kessel, & Rolston, 2006). The NGHGB was calculated as the difference between the increases in GWP due to higher direct N_2_O emissions and the decreases due to higher SOC accumulation and lower indirect N_2_O emissions under the cover crops.

### 2.3 Data analyses

We used R version 3.5.2 (R Core Team, 2018) to perform exploration, harmonization and analyses of the data. The distributions of N leaching, grain yield, N in grain, N_2_O and SOC measurements were characterized using the “fitdistrplus” package version 1.0‐14 (Delignette‐Muller & Dutang, 2015). To investigate difference on all sites where both the control and cover crop treatments (cover crop types, climatic zones, tillage systems) had N leaching, grain yield, N in grain, N_2_O and SOC measurements, we used the “glmer” method with random effect (different studies) and Gamma (link “log”) distribution (version 1.1‐19) (Bates, Mächler, Bolker, & Walker, 2015), while *p*‐values were calculated in order to confirm the significance of the relationships using the “lmerTest” package version 3.0‐1 (Kuznetsova, Brockhoff, & Christensen, 2017). The same method was performed to test whether there was a significant difference in N leaching, grain yield, N in grain, N_2_O emissions and SOC between cover crops, tillage, climatic zones and soil texture types. A linear mixed effects model was applied to investigate whether there was an effect of cover crops, tillage, climatic zones and soil texture types on physicochemical values. A linear mixed effects approach was also used to compare N leaching (%) of cover crops (legume, non‐legume and legume–non‐legume mixed), with added N fertilizer as covariate in the model. The package “akima” version 0.6‐2 was used to create interpolated contour plots (Akima, Gebhardt, Petzold, & Maechler, 2016) of pairs of the BD, pH and added N as *x*‐axis and *y*‐axis with N leaching and SOC as the *z* variable. A contour plot is a graphical technique for representing a three‐dimensional surface by plotting constant *z* slices on a two‐dimensional format. That is, given a value for *z*, lines are drawn for connecting the (*x*,*y*) coordinates where that *z* value occurs. We performed linear regressions of different variables against N leaching and SOC.

## 3 RESULTS

### 3.1 Impacts of cover crops (legume, non‐legume and legume–non‐legume mixed) on N leaching

**Figure 2:**
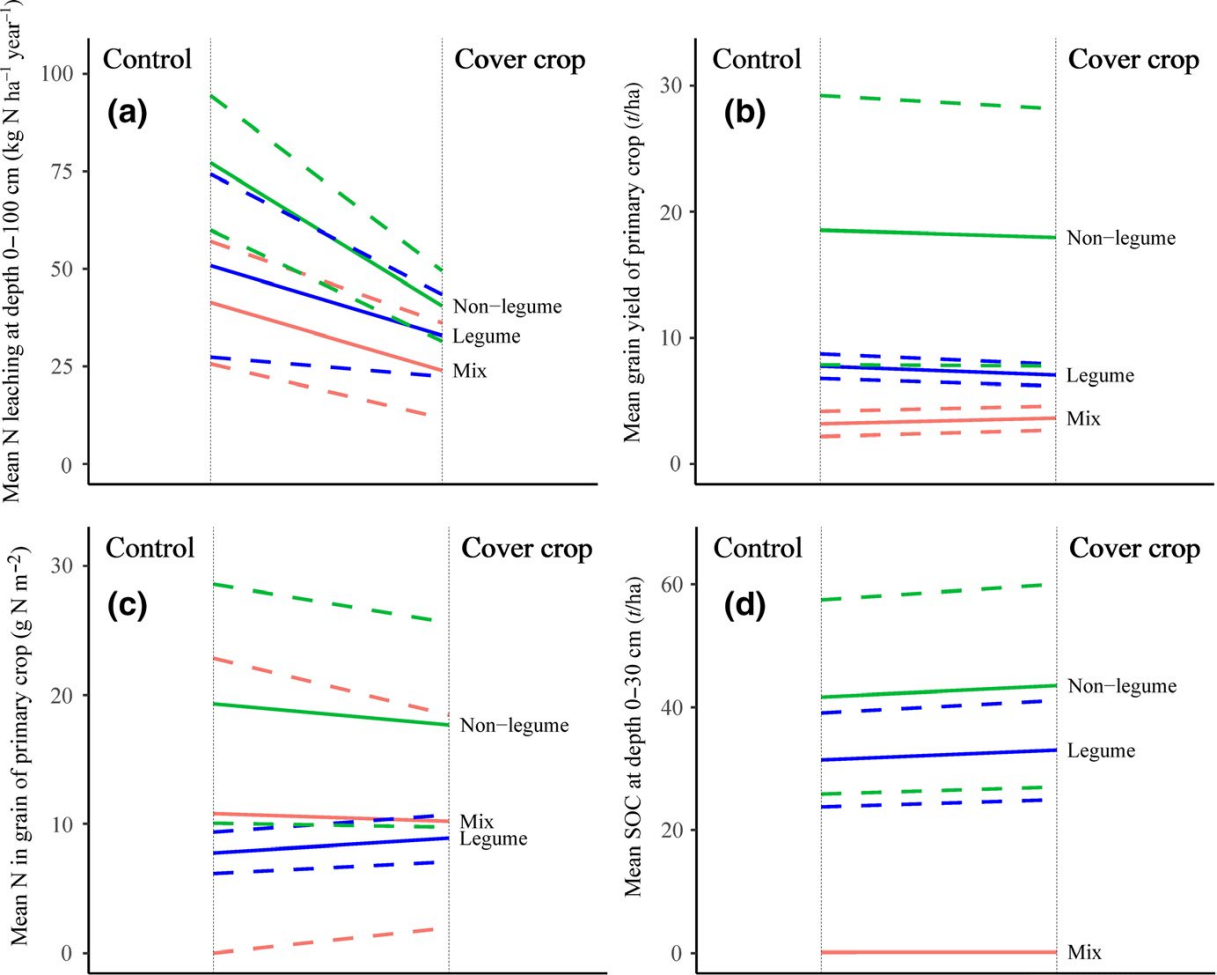
Comparisons between N leaching (a), grain yield (b), N in grain (c) and SOC (d) from control and cover crops (CC) treatments. Types of cover crops (legume [blue], non‐legume [green] or mixed [red]) and their 95% confidence intervals

**Figure 3:**
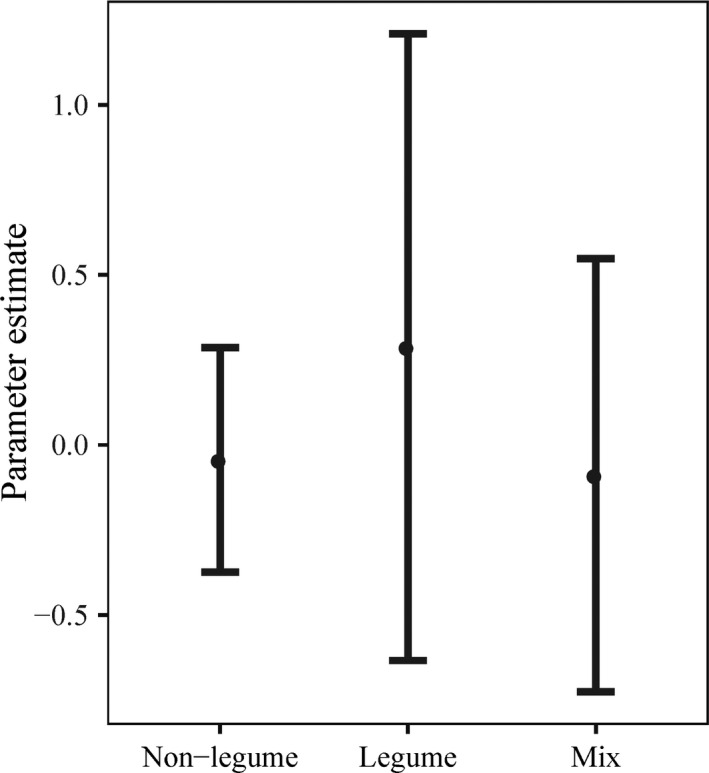
Relationships between change in N leaching (%) and legume, non‐legume and mixed cover crops. On the *y*‐axis are parameter estimates of N leaching based on a linear mixed effects model with added N fertilizer as a covariate (*n* = 66; *p* > 0.3; vertical bars denote 95% confidence intervals)

The inclusion of cover crops in the crop rotation significantly decreased N leaching compared to the control treatments (*p* < 0.001; *n* = 75). All types of cover crops had significant effects on N leaching; legume (*p* < 0.05; *n* = 11), non‐legume (*p* < 0.001; *n* = 55) and legume–non‐legume mixed cover crops (*p* < 0.001; *n* = 9) (Figure 2a). A one‐way model with random effects showed no significant (*p* > 0.05) difference in N leaching between legume, non‐legume and legume–non‐legume mixed cover crops. Additionally, a linear mixed effects model with added N fertilizer as covariate showed no significant (*p* > 0.05) effect of cover crops on the change of N leaching (%), after controlling for the effect of added N fertilizer application rate (the covariate) (Figure 3).

### 3.2 Impacts of cover crops (legume, non‐legume and legume–non‐legume mixed) on SOC and direct N_2_O emissions

A paired test with random effects showed that SOC under the cover crops was significantly higher compared to that in the control treatments (*p* < 0.001; *n* = 43). Both legume (*p* < 0.001, *n* = 29) and non‐legume (*p* < 0.001; *n* = 13) cover crops significantly increased SOC (Figure 2d). The same test showed that cover crops (*n* = 28) had no significant effect (*p* > 0.05) on direct N_2_O emissions, compared to the control treatment. Only legume (*n* = 8) cover crops significantly increased direct N_2_O emissions but non‐legume (*n* = 17) and legume–non‐legume had no effects, compared to the control treatment.

Tillage had no effect on direct N_2_O emissions. However, the changes in direct N_2_O emissions (%) under conservation tillage were significantly lower compared to that under conventional tillage treatment (Table 1).

**Table 1:** Effects of tillage on direct N_2_O emission (kg ha^−1^ year^−1^) from control and cover crop treatments

Treatment	Mean ± *SD* (conventional)	N (conventional)	Mean ± *SD* (conservation)	N (conservation)	*t*value	*p*
Control	0.94 ± 1.0	12	3.70 ± 2.74	10	−0.68	ns
Cover crops	1.46 ± 1.61	12	3.95 ± 2.91	10	0.54	ns
Change in N_2_O emissions (%)	50.58 ± 148.34	12	16.65 ± 38.94	10	4.74	*p* < 0.001

Abbreviations: N, number of observation; *SD*, standard deviation; ns, not significant.

### 3.3 Impacts of cover crops (legume, non‐legume and legume–non‐legume mixed) on grain yields and N in grain of primary crop

Overall, the cover crops significantly decreased grain yield of the primary crops compared to the control treatments (on average −4%; *p* < 0.001; *n* = 154) (Figure 2b). Both legume and non‐legume cover crops significantly decreased (*p* < 0.001; *n* = 52 and *p* < 0.01; *n* = 96 respectively) grain yield of the primary crop whilst legume–non‐legume mixed cover crops significantly increased (*p* < 0.01; *n* = 6) grain yield of the primary crop (by ≈13%). Cover crops significantly (*p* < 0.001; *n* = 118) decreased grain yield of the primary crop under conventional tillage but had no effect under conservation tillage (*n* = 20; *p* > 0.05). Overall, cover crops had no significant effect on N content in the grain of the primary crop (*p* > 0.05; *n* = 58) (Figure 2c). The legume cover crops significantly increased N in the grain of the primary crop (*p* < 0.001; *n* = 15) whilst the non‐legumes significantly decreased it (*p* < 0.05; *n* = 39). Legume–non‐legume mixed cover crops had no effects (*p* > 0.05; *n* = 4) on N in grain of the primary crop.

### 3.4 Influences of management, soil and climatic zones on cover crop efficiency to decrease N leaching and to increase SOC

**Figure 4:**
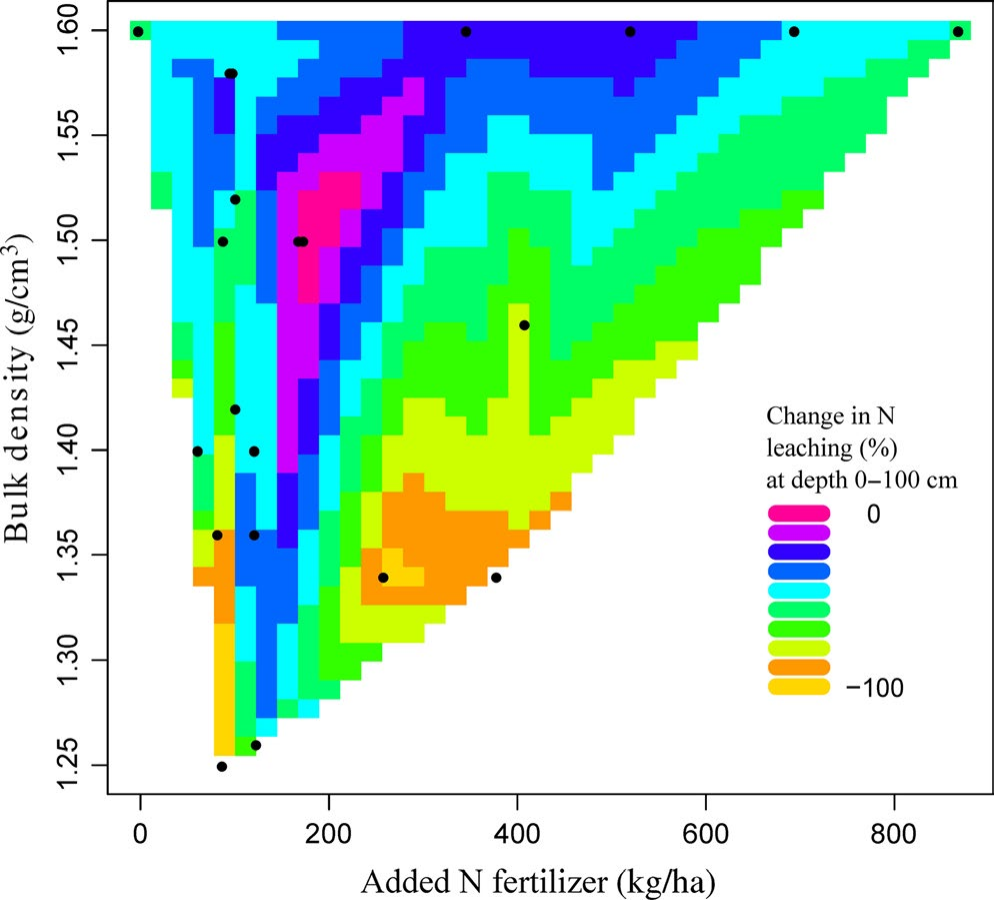
Contour plot (*n* = 38) showing relationships between added N fertilizer application rate, bulk density (BD) and change in N leaching (%) at 0–100 cm soil depth. These two variables explain 11.6% of N leaching overall variation (*p* < 0.05). N leaching significantly depended on BD (*t* = 2.62; *p* < 0.05). One outlier was removed (BD = 2.5)

**Figure 5:**
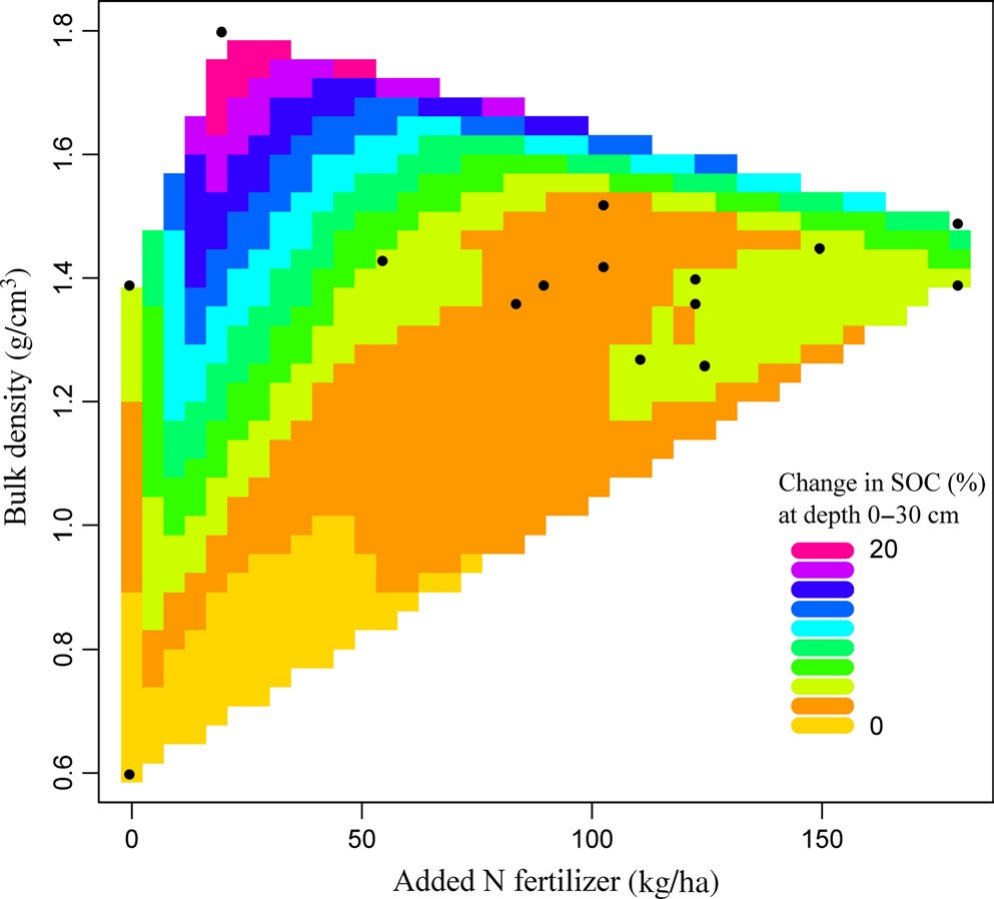
Contour plot (*n* = 41) showing relationships between added N fertilizer application rate, bulk density (BD) and change in soil organic carbon (SOC) (%). Added N fertilizer and BD explain 57% of SOC overall variation (*p* < 0.001). The SOC depended significantly on added N (*t* = −3.2; *p* < 0.01) and BD (*t* = 7.1; *p* < 0.001)

**Figure 6:**
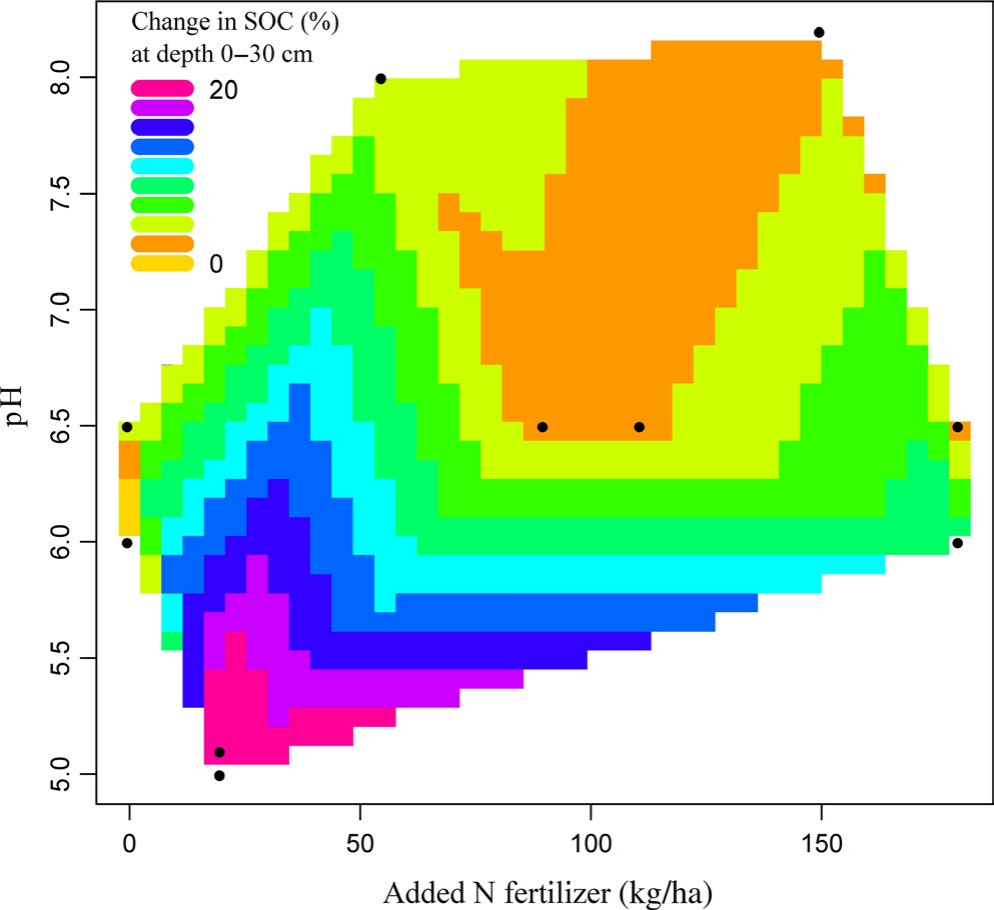
Contour plot (*n* = 35) showing relationships between added N fertilizer application rate, pH and change in soil organic carbon (SOC) (%). Added N fertilizer and pH explain 31% of SOC overall variation (*p* < 0.001). SOC depended significantly on pH (*t* = 3.94; *p* < 0.001)

For N leaching at 0–100 cm depth, contour plots based on available data showed that BD and N fertilizer application rate explained 11.6% of overall variance (*p* < 0.05; *n* = 38). N leaching was significantly related to BD (*p* < 0.05) (Figure 4). For the SOC at 0–30 cm depth, BD and N fertilizer application rate explained 57% of the overall variance in SOC (*p* < 0.001; *n* = 41). The increase in SOC under cover crops was significantly related to both N fertilizer application rate (*p* < 0.01) and BD (*p* < 0.001) (Figure 5). The interaction between soil pH and N fertilizer application rate had no significant effect on N leaching (*p* > 0.05; *n* = 43). Soil pH and added N fertilizer application rate significantly influenced SOC and explained 31% of the overall variance (*p* < 0.001; *n* = 35). However, changes in SOC varied significantly with soil pH (*p* < 0.001) (Figure 6). Soil texture had no significant (*p* > 0.05) impacts on the change in N leaching or SOC. The N leaching and SOC under the control and cover crop treatments were both not significantly (*p* > 0.05) influenced by MAAT.

**Figure 7:**
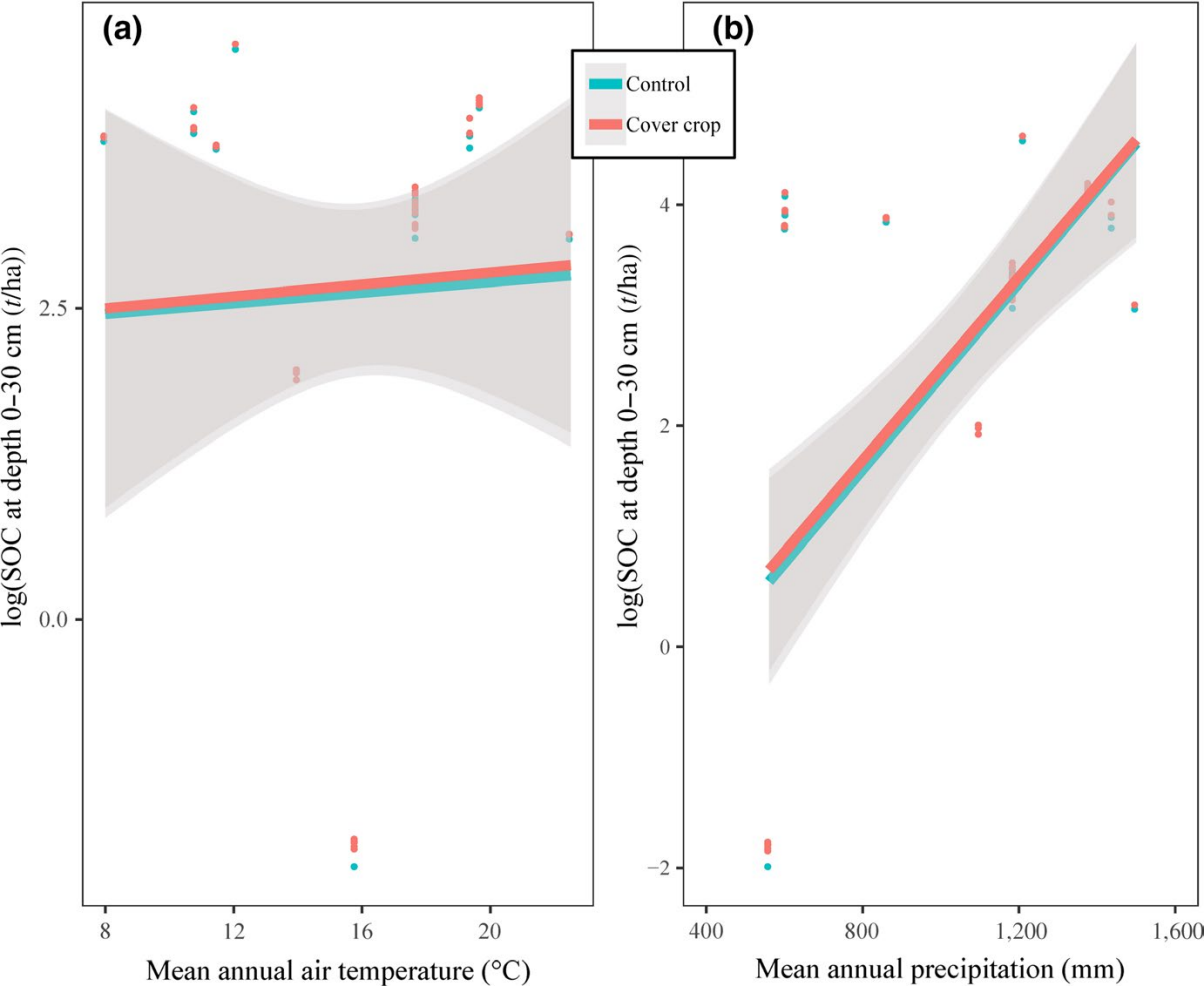
Relationships between soil organic carbon (SOC) and mean annual air temperature (MAAT) (a) and mean annual precipitation (MAP) (b) under control and cover crops. MAAT was not significantly correlated with SOC (*p* > 0.05). MAP was positively correlated with SOC for both the control (*t* = 5, *p* < 0.001; *r* ^2^ = 0.39, *p* < 0.001, *n* = 43), and cover crop (*t* = 5, *p* < 0.001; *r* ^2^ = 0.39, *p* < 0.001, *n* = 43)

Cover crops significantly decreased N leaching under both MW (*p* < 0.001; *n* = 13) and MC (*p* < 0.001; *n* = 58) climatic zones. MAP positively correlated with SOC for the control (*r* ^2^ = 0.39, *p* < 0.001; *n* = 43), and cover crop (*r* ^2^ = 0.39, *p* < 0.001; *n* = 43) treatments (Figure 7). Cover crops significantly increased SOC under MW (*p* < 0.001; *n* = 37) and under MC (*p* < 0.001; *n* = 6) climatic zones. Under both the conventional (*n* = 62) and conservation (*n* = 12) tillage systems, cover crops significantly (*p* < 0.001) decreased N leaching compared to the control. A *t* test with random effects showed that conservation tillage (*n* = 62) significantly increased N leaching for the control (*p* < 0.05) treatment compared to conventional tillage (*n* = 12). There were no significant (*p* > 0.05) effects on SOC due to tillage systems. The SOC was significantly higher under both the conventional (*p* < 0.001, *n* = 18) and conservation (*p* < 0.001, *n* = 17) tillage systems compared to the control.

### 3.5 Impacts of cover crops on net greenhouse gas balance

Cover crops increased SOC and decreased N leaching and thereby lowered the indirect N_2_O emissions (i.e. from N leaching) without significantly increasing direct N_2_O emissions. This combination of higher SOC and the lower indirect N_2_O emissions under the cover crops resulted in a lower NGHGB compared to the control treatment. The estimated reduction in NGHGB due to cover crops, compared to the control treatments, was 2.06 ± 2.10 Mg CO_2_‐eq ha^−1^ year^−1^. The reductions in NGHGB due to different cover crop types, compared to the control treatments, were 1.87 ± 1.82, 1.82 ± 1.44 and 5.15 ± 3.51 Mg CO_2_‐eq ha^−1^ year^−1^ for the legume, non‐legume and legume–non‐legume mixed cover crops respectively (Table 2). No significant difference (*p* > 0.05) was found between the different cover crop types.

**Table 2:** Descriptive statistics of the reduction in net greenhouse gas balance (NGHGB) related to the reduction of indirect nitrous oxide (N_2_O) emission and soil organic carbon sequestration (Mg CO_2_‐eq ha^−1^ year^−1^)

Type of cover crop	Change in direct N_2_O (mean ± *SD*)	Change in indirect N_2_O (mean ± *SD*)	Change in SOC (mean ± *SD*)	*N*	NGHGB (mean ± *SD*)
Legume	0.04 ± 0.05	−0.30 ± 0.37	1.61 ± 1.82	30	1.87 ± 1.82
Non‐legume	0.09 ± 0.11	−0.07 ± 0.28	5.12 ± 5.51	13	1.82 ± 1.44
Mixed	0.04 ± 0.03	−0.50 ± 0.37	0.30 ± 0.37	4	5.15 ± 3.51
All types	0.08 ± 0.10	−0.16 ± 0.33	1.97 ± 2.10	47	2.06 ± 2.10

Note

Negative numbers represent N_2_O gas emissions, while positive numbers represent gain of C by the soil.

Abbreviations: *N*, number of observations; *SD*, standard deviation.

## 4 DISCUSSION

### 4.1 Impacts of cover crops (legume, non‐legume and legume–non‐legume mixed) on N leaching

In this critical global review and systematic analysis, we found that all types of cover crops significantly decreased N leaching. However, no statistically significant differences between legume, non‐legume and legume–non‐legume mixed cover crops were found. Previous studies reported that non‐legume (Aronsson, Stenberg, & Ulén, 2011; Thomsen & Hansen, 2005; Torstensson & Aronsson, 2000), legume (Askegaard & Eriksen, 2008; Askegaard, Olesen, Rasmussen, & Kristensen, 2005; Salmerón, Cavero, Quilez, & Isla, 2010) and legume–non‐legume mixed (Askegaard, Olesen, Rasmussen, & Kristensen, 2011; Benoit, Garnier, Anglade, & Billen, 2014) cover crops can all reduce N leaching, but with different efficiencies. In the United States, Kaspar, Jaynes, Parkin, Moorman, and Singer (2012) reported that the use of non‐legume cover crops (e.g. oat and rye) is a suitable management option for reducing N leaching from corn–soybean rotations, thereby improving both water and soil quality. Non legume cover crops reduced soil NO_3_ content, which is vulnerable to N leaching during autumn and winter (Thorup‐Kristensen et al., 2003), and made additional soil N available for the primary crop following mineralization of their residues (Kaspar & Singer, 2011). In studying future scenarios over a period of 45 years, Tribouillois, Constantin, and Justes (2018) found that non‐legume cover crops continuously decreased N leaching compared to that of bare soil, but legume cover crop scenarios did not. Moreover, some simulation studies have suggested that the efficiency of legume cover crop species to reduce N leaching was about half of that of non‐legume species (e.g. Brassicaceae and Poaceae; Justes et al., 2012). Nevertheless, Valkama, Lemola, Känkänen, and Turtola (2015) reported that legume cover crops may not be effective in reducing N leaching but growing non‐legume cover crops within a spring cereal crop is an effective method for reducing N leaching from different crop varieties, soils and weather conditions. Here, it is accepted that there is a trade‐off between potential grain yield loss and environmental benefits, but this could be compensated for in environmental stewardship schemes in those countries. Leslie, Wang, Meyer, Marahatta, and Hooks (2017) recommended growing cover crops in some years only, to avoid a preemptive competition where the cover crops could recover soil NO_3_ that would otherwise have been available to the subsequent primary crop. The non‐legume cover crops can also increase N leaching when grown too late in spring or in dry areas, where the risk for N leaching is low (Thorup‐Kristensen et al., 2003). Thus, the timing and location of the non‐legume cover crops need to be considered carefully to avoid competition with the primary crop.

### 4.2 Impacts of cover crops (legume, non‐legume and legume–non‐legume mixed) on SOC and direct N_2_O emissions

Cover crops (i.e. both legume and non‐legume) increased SOC, and so they can enhance C sequestration in soils. Similar conclusions regarding the impact of cover crops on SOC were reported by Olson, Ebelhar, and Lang (2007), Poeplau and Don (2015), Wortman, Francis, Bernards, Drijber, and Lindquist (2012) and others. According to Ding et al. (2006), both organic carbon and light fraction C contents increased in soils under cover crops, with or without N fertilizer. Here, the decomposition of dead roots and biomass of cover crops results in improved SOM quantity and quality (Villamil, Bollero, Darmody, Simmons, & Bullock, 2006). This could help improve food security, reduce NGHGB and mitigate climate change.

We found that cover crops had no significant effect on direct N_2_O emissions compared to the control. According to Webb, Harrison, and Ellis (2000), cover crops increase the direct N_2_O emissions when residues are incorporated into the soil or by increasing the photosynthetically derived C supply from actively growing root systems. However, adjusting the N fertilizer application rate (e.g. by integrated soil fertility management) could help in reducing gas emissions (Guardia et al., 2016; Tribouillois et al., 2018). Previous studies reported contrasting results with regard to cover crop effects on direct N_2_O emissions (Abdalla et al., 2013; Basche et al., 2014; Mitchell, Castellano, Sawyer, & Pantoja, 2013). This could be explained by the large variations in many factors, for example cover crop types and performances, climate, soil characteristics, tillage and seasons of N_2_O samplings, between the different studies. Cover crops have the ability to decrease indirect N_2_O emissions (i.e. from N leaching). Cover crop species influence abiotic and biotic soil factors differently (Abalos, Deyn, Kuyper, & Groenigen, 2014). They have the capacity to simultaneously mitigate N leaching and indirect N_2_O emissions (Kim et al., 2015) by limiting N availability. They deplete the soil NO_3_ pool, which is the major substrate for denitrification (Liebig et al., 2015), reducing N leaching and consequently decreasing the contribution of indirect N_2_O emissions to the NGHGB. However, this depends on many factors, for example cover crop types, performances, climate, tillage and soil characteristics. In contrast, Zhou and Butterbach‐Bahl (2013) found that for coarser textured soils, the reduction in N leaching can increase availability of soil N, which can lead to a trade‐off by enhancing N_2_O emissions.

### 4.3 Influences of management, soil and climatic zones on cover crop efficiency to decrease N leaching and increase SOC

Cover crops were most efficient in reducing N leaching when the BD was <1.4 g/cm^3^ and N fertilizer application rate was >200 kg N/ha. Snapp et al. (2005) found application of more N fertilizer, especially with legume cover crops, can increase the risk of nutrient leaching, if a subsequent primary crop is not planted promptly. Thus, to reduce N leaching from soils under cover crops, judicious quantities of N fertilizer should be applied at appropriate application times, with appropriate methods (Fan, Hao, & Malhi, 2010; Yogesh & Juo, 1982). Also, to avoid losing the excess N in soils by leaching, the amount of N fertilizer applied should be based on soil and crop requirement tests (Bundy & Andraski, 2005; Defra, 2010).

In this study, we found enough data points for MW and MC climatic zones but not for DW and DC climatic zones. This is obviously because cover crops are rarely grown in dry climates as they use water that could be used to grow a primary crop and reduce water percolation by transpiration (Weinert, Pan, Moneymaker, Santo, & Stevens, 2002). Additionally, in such climates, cover crops compete with the primary crop for nutrients (Unger & Vigil, 1998) and consequently have negative impacts on crop growth and productivity. Tribouillois et al. (2018) and Wortman et al. (2012) reported that the large quantity of soil water used by the cover crops, at the cost of the subsequent primary crop and immobilization of soil N due to incorporation of low quality cover crop residues into the soil, is also a major concern. These problems appear mostly in arid and semiarid environments (<500 mm annual rainfall) where water storage in soils declines with the establishment of cover crops, and results in reduced crop yields (Cherr, Scholberg, & McSorley, 2006; Nielsen & Vigil, 2005). Conservation tillage significantly decreased the efficiency of cover crops to decrease N leaching under control treatment compared to that under conventional tillage. The large pores that can develop under conservation tillage result in high N leaching if present after broadcasting N fertilizer (CTS, 2011), and thereby could also increase GHG emissions (Smeaton, Cox, Kerr, & Dynes, 2011). Fraser et al. (2013) found that tillage had some effects on N leaching, though the use of minimum tillage for autumn cultivation resulted in significantly less N leaching than either intensive or no‐till. Buchi, Wendling, Amosse, Necpalova, and Charles (2018) reported that cover crop could maintain wheat yield and improve soil fertility and nutrient cycling in a no‐till system. Therefore, a combination of the right type of conservation tillage with cover crops could be the best management to reduce N leaching in dry climates. Water utilization by the cover crops is counterbalanced by the improved infiltration and reduced evaporative losses that occur in conservation tillage systems (Unger & Vigil, 1998; Wang & Ngouajio, 2008). Further, the high soil moisture under conservation tillage positively influences microbial activity (Madejon et al., 2009) and increases bypass flow (CTS, 2011). This could also slow the rate of mineralization, as soils take longer to warm in the spring (Abdalla et al., 2013).

We found no significant effects on the efficiency of cover crops to decrease N leaching between the MW and MC climate zones. Fraser et al. (2013) and Hooker et al. (2008) found that inter‐annual weather variability and soil types explain the variability of cover crop effectiveness in the temperate regions. Previous studies found that the effectiveness of cover crops to reduce N leaching is highly variable, both across and within different climatic zones (Quemada et al., 2013; Thorup‐Kristensen et al., 2003; Tonitto, David, & Drinkwater, 2006). In this study, soil texture had no significant impacts on N leaching under cover crops. In a review by Valkama et al. (2015), a similar relative reduction (%) in N leaching losses by cover crops, compared to the controls, across different soil textures in the Nordic countries was reported. By contrast, Premrov, Coxon, Hackett, Kirwan, and Richards (2014) concluded that, under mild temperate winter conditions, the risk of N leaching from light textured, freely draining soils is high and therefore, it is important to establish over‐winter cover crops. In the driest parts of south‐east England, early sown cover crops were found to be most effective on freely drained sandy soils, where the risk of N leaching was high, but were less effective on medium to heavy textured soils with poorer drainage (Macdonald, Poulton, How, Goulding, & Powlson, 2005).

Under cover crops, soils with higher BD are the most likely to have higher SOC. The presence of N in soil is important for SOC accumulation as C sequestration requires N (van Groenigen et al., 2017). According to Aula, Macnack, Jeremiah, Mullock, and Raun (2016), the use of N fertilizer significantly increases SOC. The difference in SOC (%) between the cover crops and the control treatments was at its highest at low N fertilizer rate. High soil pH decreases the efficiency of cover crops to accumulate SOC. Parfitt, Timm, Reichardt, and Pauletto (2014) reported that high pH (due to liming) possibly reduces SOC. Both soil texture and tillage had no significant impacts on the efficiency of cover crops to sequester SOC, compared to control treatments. Previous studies showed both beneficial (Gonzalez‐Sanchez, Ordonez‐Fernandez, Carbonell‐Bojollo, Veroz‐Gonzalez, & Gil‐Ribes, 2012; West & Post, 2002) and no impact (Dimassi et al., 2014; Powlson et al., 2014) of no‐till relative to conventional tillage on SOC. Soil organic matter and organic residues are the two main energy sources of microbial biomass (Brookes et al., 2008). Higher SOC is advantageous for soil fertility, water holding capacity and nutrient retention and therefore is considered essential for sustainable agriculture (Hoyle, 2013).

### 4.4 Impacts of cover crops (legume, non‐legume and legume–non‐legume mixed) on grain yield and N content in grain of the primary crop

We found, overall, cover crops decreased grain yields of the primary crop by ≈4% compared to the control treatment. Both legume and non‐legume cover crops decreased grain yields but legume–non‐legume mixed cover crops increased yield significantly (by ≈13%). Studies found that grain yields of the primary crop can be improved by incorporation of legume–non‐legume mixtures (Doltra & Olesen, 2013) or legume (Campiglia et al., 2011) cover crops. A review by Tonitto et al. (2006) reported a 10% reduction in grain yield of primary crops under legume cover crops. In contrast, Coombs, Lauzon, Deen, and Eerd (2017) found alfalfa and red clover (legume) had a positive impact on corn yield in 1 of 2 years. Dozier, Behnke, Davis, Nafziger, and Villamil (2017) and Marcillo and Miguez (2017) found non‐legume cover crops had no effects on the grain yield of corn, especially in the short term. Noland et al. (2018) found that to reduce soil NO_3_ while maintaining corn and subsequent soybean yields, cover crops should be inter‐seeded into corn at the seven‐leaf collar stage. Nevertheless, a successful termination for the cover crops is crucial to avoid competition with the subsequent soybean crop. The legume cover crops increased N in the grain of the primary crop, while non‐legumes decreased it and legume–non‐legume mixed cover crops had no significant effect. Wittwer, Dorn, Jossi, and Heijden (2017) found higher grain N concentrations and N contents under both legume and legume–non‐legume mixed cover. However, there are mixed results concerning the effects of cover crops on N content in grain of the primary crop in the literature (Doltra & Olesen, 2013; Kramberger, Gselman, Janzekovic, Kaligaric, & Bracko, 2009; Olesen, Hansen, Askegaard, & Rasmussen, 2014; Rinnofner et al., 2008; Thomsen, 2014).

### 4.5 Impacts of cover crops (legume, non‐legume and legume–non‐legume mixed) on net greenhouse gas balance

Characterising the effects of cover crops on the NGHGB of cropping systems is complex given that they influence both the carbon balance as well as direct and indirect N_2_O emissions. The uncertainty in our results, due to assumptions made, was conservatively estimated by calculating the standard deviations for all values. Our study showed that all cover crop types could contribute to ecological intensification and climate change mitigation by improving the NGHGB, compared to the control treatment. Cover crop practices could also contribute to the aspirations of the soil C “4‐per‐mille” initiative (Minasny et al., 2017), especially in wet regions where C stocks are low and nutrients are available (e.g. North Europe, North China and Canada). The growing cover crops could increase water use, keeping soils dry and thereby reduce rates of SOC decomposition, as well as reducing N_2_O loss and soil erosion (Desjardins, Smith, Grant, Campbell, & Riznek, 2005). In contrast, Negassa, Price, Basir, Snapp, and Kravchenko (2015) reported that the addition of cover crop inputs to topographic depression areas can increase the priming effect (Guenet, Neill, Bardoux, & Abbadie, 2010), which increases decomposition of native SOC, and thereby increases CO_2_ emissions, when stimulated by additions of fresh plant residue inputs. However, Steele, Coale, and Hill (2012) reported no changes in organic matter content after 13 years of a cover crop experiment. One limitation of our analysis is that the majority of the studies collected were short‐term experiments (2–3 years). Berntsen, Olesen, Petersen, and Hansen (2006) reported that the effects of cover crops should be evaluated in the long term rather than considering short‐term effects only; however, there is a scarcity of such long‐term experiments. We found that incorporating cover crops, specifically legume–non‐legume mixed cover crops, into the crop rotation is beneficial for soils, the environment and crop productivity. Tonitto et al. (2006) found that the legume–non‐legume mixed cover crops were useful for both atmospheric N_2_ fixation and for soil residual nitrate recycling. Cover crops influence soil N and C dynamics and N available for the subsequent primary crop. They play an important role in achieving more diverse and multifunctional agricultural systems (Blanco‐Canqui et al., 2015; Schipanski et al., 2014), suggesting that further efforts are required to enable farmers to overcome all barriers for their widespread adoption (Roesch‐McNally et al., 2017). However, management practices in relation to cover crops will need to be adapted to specific soil, management and regional climatic conditions.

## 5 CONCLUDING REMARKS

This critical global review and systematic analysis reveals that, by adopting cover crops, we could decrease N leaching to ground water and increase SOC sequestration without having significant effects on direct N_2_O emissions. To avoid the negative impacts of cover crops on grain yield (−4%), legume–non‐legume mixed cover crops, which increase the yield by ≈13% and had no significant impacts on N in grain, should be selected. Overall, cover crops can mitigate NGHBG by 2.06 ± 2.10 Mg CO_2_‐eq ha^−1^ year^−1^. These effects can be considered important in contributing to the resilience of farming systems to environmental changes, for example from climate change, by being more fertile, productive and have better water quality. However, to increase the effectiveness of cover crops, field management techniques should be optimized to the local climatic conditions, water resources, soil and cropping systems. The genetics of cover crop species could be improved to provide deeper rooted crops, which have higher N use efficiencies, better nitrate scavenging abilities and lower N leaching potential. Deep rooted species could help with cover crop resilience, for example deeper delivery of C in the soil profile. It is also important to adjust timings and dates of the planting and kill of the cover crops, to avoid competition with the primary crop, to improve their effectiveness and avoid trying to establish cover crops when soil conditions are suboptimal (potentially increasing soil erosion losses). Although cover crops increase costs, due to the need to purchase new seeds, management operations and termination costs, these costs can be compensated for if the wider benefits are considered. These include retention and carryover of nutrients between phases of a rotation, and the opportunity for the cover crops to be sold as forage or grazed. A positive return from cover crops for producers is a possibility, especially if they replace a fallow period instead of a primary crop. However, to support the widespread adoption of cover crops, improved policy, education, training and awareness raising of the potential benefits and risks and risk abatement strategies are needed.
