# Supplementary material for: A critical review of the impacts of cover crops on nitrogen leaching, net greenhouse gas balance and crop productivity
Source: Glob Chang Biol. 2019 May 13;25(8):2530–43. doi: 10.1111/gcb.14644 (PMC6851768; doi:10.1111/gcb.14644)
Supplement: Supplementary file 1 [file GCB-25-2530-s001.docx]

Table 1: Published studies on the impacts of cover crops, climate and soil properties on N leaching and indirect N_2_O emissions at the top 100 cm soil depth.

| Location  (country/ state) | MAAT  (^o^C) | | MAP  (mm) | | Soil  texture | Climatic zone | BD  (g cm^-3^) | pH^a^ | Tillage | Primary crop (C) | Cover crops (CC) | Type of cover crops | Added N ( kg N ha^-1^y^-1^) | Duration  (year) | Original depth  (cm) | N leach for C ( 0-100cm; kg N ha^-1^ y^-1^)¶ | N leach for CC  ( 0-100cm; kg N ha^-1^ y^-1^)¶ | ∆ indirect N_2_O emissions due to CC (kg N ha^-1^y^-1^)** | Ref. |
| --- | --- | --- | --- | --- | --- | --- | --- | --- | --- | --- | --- | --- | --- | --- | --- | --- | --- | --- | --- |
| Mellby, SE | | 7.2 | 803 | | Sandy loam | MC | ND | ND | R | Spring barley | Red clover/ryegrass | M | 90 | 2 | 90 | 18.1 | 12.8 | 0.0 | 1 |
| Mellby, SE | | 7.2 | 803 | | Sandy loam | MC | ND | ND | Con | Cereals/potatoes | Perennial ryegrass | NL | 97 | 5 | 90 | 62.9 | 25.6 | -0.3 | 1 |
| Mellby, SE | | 7.2 | 803 | | Sandy loam | MC | ND | ND | Con | Cereals/potatoes | Perennial ryegrass | NL | 161 | 5 | 90 | 72.5 | 28.8 | -0.3 | 1 |
| Mellby, SE | | 7.2 | 803 | | Sandy loam | MC | ND | ND | Con | Cereals/potatoes | Perennial ryegrass | NL | 263 | 5 | 90 | 85.3 | 58.7 | -0.2 | 1 |
| Mellby, SE | | 7.2 | 773 | | Sandy loam | MC | 1.6 | 5.9 | Con | Spring barley | Perennial ryegrass | NL | 97 | 6 | 90 | 18.1 | 12.8 | -0.1 | 2 |
| Southeast Finland, FIN | | ND | 650 | | Clay soil | MC | ND | 6.3 | Con | Spring barley | Italian ryegrass | NL | 90 | 5 | 90 | 12.9 | 6.1 | -0.1 | 3 |
| Southeast Finland, FIN | | ND | 650 | | Silt soil | MC | ND | 6.3 | Con | Spring barley | Italian ryegrass | NL | 90 | 5 | 90 | 7.5 | 4.8 | 0.0 | 3 |
| Southeast Finland, FIN | | ND | 650 | | Sandy soil | MC | ND | 6.3 | Con | Spring barley | Italian ryegrass | NL | 90 | 5 | 90 | 27.3 | 9.7 | -0.1 | 3 |
| Southeast Finland, FIN | | ND | 650 | | Peat soil | MC | ND | 6.3 | Con | Spring barley | Italian ryegrass | NL | 45 | 5 | 90 | 32.9 | 23.5 | -0.1 | 3 |
| Lincoln, NZ | | ND | ND | | Silt loam | MW | 1.25 | 5.6 | Con | Bare soil | Ryegrass | NL | 89 | 2 | 25 | 41.7 | 3.2 | -0.3 | 4 |
| Canterbury, NZ | | 11.4 | 680 | | Silt loam | MW | 1.4 | 5.4 | IT | barley; wheat | Forage rape | NL | 63 | 7 | 60 | 237.7 | 106.3 | -1.0 | 5 |
| Canterbury, NZ | | 11.4 | 680 | | Silt loam | MW | 1.4 | 5.4 | RT | barley; wheat | Forage rape | NL | 63 | 7 | 60 | 219.4 | 83.4 | -1.0 | 5 |
| Canterbury, NZ | | 11.4 | 680 | | Silt loam | MW | 1.4 | 5.4 | No-till | barley; wheat | Forage rape | NL | 63 | 7 | 60 | 228.6 | 141.7 | -0.7 | 5 |
| Canterbury, NZ | | 11.4 | 680 | | Silt loam | MW | 1.4 | 5.4 | IT | Peas | Forage rape | NL | 63 | 7 | 60 | 237.7 | 106.3 | -1.0 | 5 |
| Canterbury, NZ | | 11.4 | 680 | | Silt loam | MW | 1.4 | 5.4 | RT | Peas | Forage rape | NL | 63 | 7 | 60 | 219.4 | 83.4 | -1.0 | 5 |
| Canterbury, NZ | | 11.4 | 680 | | Silt loam | MW | 1.4 | 5.4 | NT | Peas | Forage rape | NL | 63 | 7 | 60 | 228.6 | 141.7 | -0.7 | 5 |
| Carlow, IRE | | 9.4 | 824 | | Coarse sandy loam | MC | ND | ND | R | Barley | Mustard | NL | 151 | 3 | 90 | 87.3 | 25.1 | -0.5 | 6 |
| Carlow, IRE | | 9.4 | 824 | | Coarse sandy loam | MC | ND | ND | Con | Barley | Mustard | NL | 151 | 3 | 90 | 81.6 | 51.0 | -0.2 | 6 |
| Jiangsu, CN | | 15.7 | ND | | Loam | MW | 1.6 | 5.6 | Con | Tomato, Sweet, corn, Cucumber, Celery | Sweet corn | NL | 0 | 2 | 50 | 16.0 | 7.4 | -0.1 | 7 |
| Jiangsu, CN | | 15.7 | ND | | Loam | MW | 1.6 | 5.6 | Con | Tomato, Sweet, corn, Cucumber, Celery | Sweet corn | NL | 348 | 2 | 50 | 35.4 | 23.4 | -0.1 | 7 |
| Jiangsu, CN | | 15.7 | ND | Loam | | MW | 1.6 | 5.6 | Con | Tomato, Sweet, corn, Cucumber, Celery | Sweet corn | NL | 522 | 2 | 50 | 49.1 | 34.3 | -0.1 | 7 |
| Jiangsu, CN | | 15.7 | ND | | Loam | MW | 1.6 | 5.6 | Con | Tomato, Sweet, corn, Cucumber, Celery | Sweet corn | NL | 696 | 2 | 50 | 104.6 | 54.9 | -0.4 | 7 |
| Jiangsu, CN | | 15.7 | ND | | Loam | MW | 1.6 | 5.6 | Con | Tomato, Sweet, corn, Cucumber, Celery | Sweet corn | NL | 870 | 2 | 50 | 124.6 | 58.3 | -0.5 | 7 |
| California, USA | | ND | 396 | | Fine loamy, mixed | DW | 1.46 | 7.7 | Con | Broccoli | phacelia (Phacelia tanacetifolia cv. 'Phaci') | NL | 410 | 1 | 75 | 28.7 | 7.8 | -0.2 | 8 |
| California, USA | | ND | 396 | | Fine loamy, mixed | DW | 1.46 | 7.7 | R | Broccoli | Merced rye (Secale cereale cv. 'Merced') | NL | 410 | 1 | 75 | 28.7 | 8.2 | -0.2 | 8 |
| South Jutland, DK | | 7.6 | 862 | | Sandy loam | MC | ND | ND | Con | Spring barley | Rye grass | NL | 110 fert | 3 | 20 | 69.5 | 27.3 | -0.3 | 9 |
| South Jutland, DK | | 7.6 | 862 | | Sandy loam | MC | ND | ND | Con | Spring barley | Rye grass | NL | 110 org | 3 | 20 | 69.5 | 34.8 | -0.3 | 9 |
| South Jutland, DK | | 7.6 | 862 | | Sandy loam | MC | ND | ND | Con | Spring barley | Rye grass | NL | 165 org | 3 | 20 | 69.5 | 47.2 | -0.2 | 9 |
| South Jutland, DK | | 7.6 | 862 | | Sandy loam | MC | ND | ND | Con | Spring barley | Rye grass | NL | 0 | 3 | 20 | 46.3 | 16.6 | -0.2 | 9 |
| Foulum, DK | | 7.3 | 627 | | Coarse sand | MC | 1.5 | ND | Con | Winter wheat | Oil radish | NL | 170 | 2 | 100 | 101.4 | 92.4 | -0.1 | 10 |
| Foulum, DK | | 7.3 | 627 | | Coarse sand | MC | 1.5 | ND | Con | Winter wheat | White mustard | NL | 170 | 2 | 100 | 101.4 | 89.2 | -0.1 | 10 |
| Foulum, DK | | 7.3 | 627 | | Coarse sand | MC | 1.5 | ND | Con | Spring barley | Oil radish | NL | 90 | 2 | 100 | 85.6 | 69.2 | -0.1 | 10 |
| Foulum, DK | | 7.3 | 627 | | Coarse sand | MC | 1.5 | ND | Con | Spring barley | Rye grass (autumn) | NL | 90 | 2 | 100 | 85.6 | 61.4 | -0.2 | 10 |
| Foulum, DK | | 7.3 | 627 | | Coarse sand | MC | 1.5 | ND | Con | Spring barley | Rye grass (autumn) | NL | 90 | 2 | 100 | 85.6 | 63.1 | -0.2 | 10 |
| Foulum, DK | | 7.3 | 627 | | Sandy loam | MC | 1.5 | ND | Con | Winter wheat | White mustard | NL | 170 | 2 | 100 | 63.1 | 37.8 | -0.2 | 10 |
| Foulum, DK | | 7.3 | 627 | | Sandy loam | MC | 1.5 | ND | Con | Winter wheat | Oil radish | NL | 170 | 2 | 100 | 63.1 | 58.0 | 0.0 | 10 |
| Foulum, DK | | 7.3 | 627 | | Sandy loam | MC | 1.5 | ND | Con | Spring barley | Rye grass (winter) | NL | 90 | 2 | 100 | 43.6 | 19.6 | -0.2 | 10 |
| Foulum, DK | | 7.3 | 627 | | Sandy loam | MC | 1.5 | ND | Con | Spring barley | Rye grass (winter) | NL | 90 | 2 | 100 | 43.6 | 22.2 | -0.2 | 10 |
| Foulum, DK | | 7.3 | 627 | | Sandy loam | MC | 1.5 | ND | Con | Spring barley | Oil radish | NL | 90 | 2 | 100 | 43.6 | 6.6 | -0.3 | 10 |
| Västergötland, SE | | 6.0 | 650 | | Sandy loam | MC | ND | 6.4 | Con | Winter seed rape | Rye grass | NL | 100 | 1 | 90 | 40.5 | 40.5 | 0.0 | 11 |
| Västergötland, SE | | 6.0 | 650 | | Sandy loam | MC | ND | 6.4 | Con | Peas | Rye grass | NL | 0 | 1 | 90 | 67.2 | 51.2 | -0.1 | 11 |
| Jyndevad, DK | | ND | 859 | | Coarse sandy | MC | ND | 5.5-5.9 | Con | Spring barley | Grass | NL | 70 | 3 | 100 | 98.0 | 39.0 | -0.4 | 12 |
| Jyndevad, DK | | ND | 859 | | Coarse sandy | MC | ND | 5.5-5.9 | Con | Spring barley | Clover | L | 0 | 3 | 100 | 98.0 | 45.5 | -0.4 | 12 |
| Boigneville, FR | | 11.5 | 604 | | Loamy sand/coarse | MC | 1.42 | ND | Con | Winter wheat | White mustard | NL | 103 | 16 | 90 | 30.9 | 19.2 | -0.1 | 13 |
| Boigneville, FR | | 11.5 | 604 | | Loamy sand/coarse | MC | 1.52 | ND | NT | Winter wheat | White mustard | NL | 103 | 16 | 90 | 22.4 | 14.9 | -0.1 | 13 |
| Kerlavic, FR | | 12.1 | 1213 | | Loamy sand/sandy loam | MC | 1.26 | ND | Con | Winter wheat | Italian Ryegrass | NL | 125 | 13 | 90 | 75.7 | 41.6 | -0.3 | 13 |
| Thibie, FR | | 10.8 | 605 | | Loam | MC | 1.36 | ND | Con | Winter wheat | Radish or winter cereal | NL | 84 | 13 | 90 | 33.1 | 13.9 | -0.1 | 13 |
| Thibie, FR | | 10.8 | 605 | | Loam | MC | 1.4 | ND | Con | Winter wheat | Radish or winter cereal | NL | 123 | 17 | 90 | 30.9 | 11.7 | -0.1 | 13 |
| Thibie, FR | | 10.8 | 605 | | Loam | MC | 1.36 | ND | Con | Winter wheat | Radish or winter cereal | NL | 123 | 17 | 90 | 38.4 | 16.0 | -0.2 | 13 |
| Jyndevad, DK | | ND | 859 | | Coarse sand | MC | ND | 2-4.2 | Con | Barley, wheat, grass | Ley; clover; lupin | L | ND | 4 | 80 | 117.0 | 61.8 | -0.4 | 14 |
| Jyndevad, DK | | ND | 859 | | Coarse sand | MC | ND | 2-4.2 | Con | Barley, wheat, grass | Ley; clover; lupin | L | ND | 4 | 80 | 114.8 | 61.8 | -0.4 | 14 |
| Foulum, DK | | ND | 626 | | Loamy sand | MC | ND | 2-4.2 | Con | Barley, wheat, grass | Ley; clover; lupin | L | ND | 4 | 100 | 54.0 | 38.0 | -0.1 | 14 |
| Foulum, DK | | ND | 626 | | Loamy sand | MC | ND | 2-4.2 | Con | Barley, wheat, grass | Ley; clover; lupin | L | ND | 4 | 100 | 35.0 | 26.0 | -0.1 | 14 |
| Flakkebjerg, DK | | ND | 559 | | Sandy loam | DC | ND | 2-4.2 | Con | Barley, wheat, grass | Ley; clover; lupin | L | ND | 4 | 100 | 37.0 | 39.0 | 0.0 | 14 |
| Flakkebjerg, DK | | ND | 559 | | Sandy loam | DC | ND | 2-4.2 | Con | Barley, wheat, grass | Ley; clover; lupin | L | ND | 4 | 100 | 29.0 | 28.0 | 0.0 | 14 |
| Canterbury Plains, NZ | | 11.4 | 640 | | ND | MW | 1.5 | ND | ND | Silage corn | Forage wheat | NL | 175 | 3 | 90 | 89.6 | 76.8 | -0.1 | 15 |
| South east France, FR | | 11.8 | 1514 | | Clay loam | MC | ND | ND | Con | Winter wheat | Black medic | L | 0 | 2 | 90 | 18.7 | 16.6 | 0.0 | 16 |
| South east France, FR | | 13.9 | 1840 | | Say loam | MC | ND | ND | Con | Winter wheat | Alfalfa | L | 0 | 2 | 90 | 18.7 | 17.4 | 0.0 | 16 |
| South east France, FR | | 13.9 | 1802 | | Say loam | MC | ND | ND | Con | Winter wheat | Red clover | L | 0 | 2 | 90 | 18.7 | 13.7 | 0.0 | 16 |
| South east France, FR | | 10.9 | 1147 | | Silt loam | MC | ND | ND | Con | Winter wheat | White clover | L | 0 | 2 | 90 | 18.7 | 13.7 | 0.0 | 16 |
| South-west Sweden, SE | | 7.2 | 803 | | Say loam soil | MC | ND | ND | R | Spring barley | Red clover/perennial ryegrass | M | 90 | 2 | 90 | 18.1 | 17.1 | 0.0 | 17 |
| Mellby, SE | | 7.5 | 736 | | Say loam soil | MC | 1.5 | ND | Con | Barley | Italian ryegrass | NL | 90 | 1 | 90 | 4.2 | 1.2 | 0.0 | 18 |
| Mellby, SE | | 7.5 | 736 | | Say loam soil | MC | 1.5 | ND | Con | Oat | Italian ryegrass | NL | 90 | 1 | 90 | 4.7 | 0.3 | 0.0 | 18 |
| Mellby, SE | | 7.5 | 736 | | Say loam soil | MC | 1.5 | ND | Con | Wheat | Italian ryegrass | NL | 90 | 1 | 90 | 2.9 | 0.5 | 0.0 | 18 |
| Mellby, SE | | 7.5 | 736 | | Say loam soil | MC | 2.5 | ND | Con | Barley | Italian ryegrass | NL | 90 | 1 | 90 | 3.3 | 4.1 | 0.0 | 18 |
| Jyevad, DK | | ND | 1091 | | Coarse sand | MC | ND | ND | Con | Spring barley, grass-clover, winter cereals | Mixed catch crop | M | 80 | 4 | ND | 91.3 | 66.2 | -0.2 | 19 |
| Foulum, DK | | ND | 823 | | Loamy sa | MC | ND | ND | Con | Spring barley, grass-clover, winter cereals | Mixed catch crop | M | 100 | 4 | ND | 41.0 | 33.8 | -0.1 | 19 |
| Flakkebjerg, DK | | ND | 721 | | Say loam | MC | ND | ND | Con | Spring barley, grass-clover, winter cereals | Mixed catch crop | M | 100 | 4 | ND | 27.7 | 19.3 | -0.1 | 19 |
| Seine Basin, FR | | 10.0 | 759 | | Loamy soil | MC | ND | ND | Con | Winter wheat, corn, faba beans | Mixed catch crop | M | 74-238 | 2 | 90 | 68.3 | 34.1 | -0.3 | 20 |
| Seine Basin, FR | | 10.0 | 759 | | Organic soil | MC | ND | ND | Con | Winter wheat, corn, faba beans | Mixed catch crop | M | 8-200 | 5 | 90 | 36.5 | 14.9 | -0.2 | 20 |
| Mellby, SE | | 7.2 | 951 | | Say loam soil | MC | 1.58 | 5.9 | Con | Spring barley | Mixed catch crop | M | 100 | 2 | 90 | 29.9 | 14.1 | -0.1 | 21 |
| Beijing, CN | | 11.0 | 635 | | Loam | MC | 1.34 | 7.81 | Con | Three vegetable | Sweet corn | NL | 380 | 3 | 90 | 197.1 | 39.5 | -1.2 | 22 |
| Beijing, CN | | 11.0 | 635 | | Loam | MC | 1.34 | 7.81 | Con | Three vegetable | Sweet corn | NL | 260 | 3 | 90 | 161.9 | 23.9 | -1.0 | 22 |
| Jiangsu, CN | | 14.0 | 1100 | | Clay | MC | ND | 6.04 | Con | Lettuce | Sweet corn | NL | 350 (U) + 70 (org) | 1 | 50 | 59.8 | 41.6 | -0.1 | 23 |
| Jiangsu, CN | | 14.0 | 1100 | | Clay | MC | ND | 6.04 | Con | Lettuce | Sweet corn | NL | 420 (U) + 70 (org) | 1 | 50 | 45.9 | 30.3 | -0.1 | 23 |
| Lanna, SE | | 6.1 | 558 | | Clay | MC | ND | 6.6 | Con | Spring barley | Perennial ryegrass | NL | 105 | 2 | 90 | ND* | ND | -0.1 | 24 |
| Lanna, SE | | 6.1 | 558 | | Clay | MC | ND | 6.6 | Con | Spring Oat | Perennial ryegrass | NL | 105 | 2 | 90 | ND* | ND | -0.1 | 24 |
| Odum, DK | | 0 | 630 | | Coarse clay loam | MC | ND | ND | Con | Spring barley | Italian ryegrass | NL | 120-135 | 5 | 90 | ND* | ND | -0.1 | 25 |
| Jyevad, DK | | 0.6 | 558 | | Clay loam | MC | ND | ND | Con | Spring barley | Italian ryegrass | NL | 120-135 | 4 | 90 | ND* | ND | -0.1 | 25 |
| Jyevad, DK | | 7.9 | 858 | | Coarse sand | MC | ND | 6.3-6.5 | Con | Green barley | Italian ryegrass | NL | 0-120 (slurry) | 1 | 70 | ND* | ND | -2.0 | 26 |
| Jyevad, DK | | 7.9 | 858 | | Coarse sand | MC | ND | 6.3-6.6 | Con | Barley | Perennial ryegrass | NL | 0-120 (slurry) | 1 | 70 | ND* | ND | -0.5 | 26 |
| Jyevad, DK | | 7.9 | 859 | | Loamy sand | MC | ND | 6.04 | Con | Spring barley/spring wheat | Perennial ryegrass/Italian ryegrass | NL | 135 | 28 | 80 | ND* | ND | 0.1 | 27 |

MAAT - mean annual air temperature (^o^C) and MAP - mean annual precipitation. * = difference in N leaching between control and cover crop was available. **= differences in indirect N_2_O emissions between the control and cover crop treatments; indirect N_2_O emissions resulting from N leaching under control and cover crop treatments were estimated using Tier I IPCC protocol (IPCC, 2006) for estimating indirect N_2_O emissions from N leaching prescribes multiplying the mass of N leached by a default factor of 0.0075 kg N_2_O-N kg^−1^. ^a^Different methods were used to measure soil pH using pH probe/ meter in deionized water or 0.01 M CaCl_2_ in 1:1 and 1:2, or 1:5 (v: v) soils: solution ratios. ND= no data available; MC= moist cool; MW= moist warm; DW= dry warm; DC= dry cool climate zone. Added N fertilizer is in kg N ha^-1^. ¶= N leaching at the top 100 cm was calculated using the depth distributions method (Jobbagy & Jackson, 2001). Con= conventional; R= reduced; NT= no-till; IT= intensive tillage; Org= organic; U= urine; L= legume; NL= non-legume and M= mixed. DK= Denmark; CN= China; SE= Sweden; IRE= Ireland; NZ= New Zealand; FR= France; USA= United States of America; ES= Spain; FIN= Finland. Ref.: 1=Torstensson &Aronsson (2000); 2=Torstensson et al. (2006); 3= Lemola and Turtola (2000);4= McLenaghen et al. (1996); 5= Fraser et al. (2013); 6=Hooker et al. (2008); 7= Min et al., (2011); 8= Wyland et al. (1996); 9=Thomsen (2005); 10=Thomsen & Hansen (2014); 11= Engstrom et al. (2011); 12= Askegaard and Eriksen (2008); 13=Constantin et al. (2010);14= Askegaard et al. (2005); 15= Teixeira et al. (2016); 16= Amosse et al. (2014); 17= Aronsson et al. (2015); 18= Lewan (1994); 19= Askegaard et al. (2011); 20= Benoit et al. (2014); 21= Bergström and Jokela (2001); 22= Liang et al.(2016); 23= Lu et al. (2013); 24= Aronsson et al. (2011); 25= Hansen a Djurhuus (1997); 26= Hansen et al. (2007); 27= Berntsen et al. (2006).
