# Supplementary material for: A critical review of the impacts of cover crops on nitrogen leaching, net greenhouse gas balance and crop productivity
Source: Glob Chang Biol. 2019 May 13;25(8):2530–43. doi: 10.1111/gcb.14644 (PMC6851768; doi:10.1111/gcb.14644)
Supplement: Supplementary file 2 [file GCB-25-2530-s002.docx]

Table 2: Published studies on the impacts of cover crops, climate and soil properties on the grain yield of the primary crop.

| Location  (country/state) | MAAT  (^o^C) | | MAP  (mm) | Soil  texture | BD  (g cm^-3^) | pH^a^ | Tillage | Primary crop (C ) | Cover crops (CC) | Type of CC | Added N (kg ha^-1^) | Duration  (year) | | Grain yield (control) (t ha^-1^) | Grain yield under (CC) (t ha^-1^) | Change in grain yield (∆G) (t ha^-1^) | Ref. |
| --- | --- | --- | --- | --- | --- | --- | --- | --- | --- | --- | --- | --- | --- | --- | --- | --- | --- |
| Mellby, SE | | 7.2 | 803 | Sandy loam soil | ND | ND | Con | Cereals; potatoes | perennial ryegrass | NL | 97 | | 5 | 5.94 | 4.52 | -1.42 | 1 |
| Mellby, SE | | 7.2 | 803 | Sandy loam soil | ND | ND | Con | Cereals; potatoes | perennial ryegrass | NL | 48 + 113 (manure) | | 6 | 6.57 | 5.14 | -1.43 | 1 |
| Mellby, SE | | 7.2 | 803 | Sandy loam soil | ND | ND | Con | Cereals; potatoes | perennial ryegrass | NL | 49 + 214 (manure) | | 7 | 5.97 | 4.97 | -1.00 | 1 |
| Mellby, SE | | 7.2 | 773 | Sandy loam soil | 1.58 | 5.9 | Con | Spring barley (other cereals and forage) | perennial ryegrass | NL | 97 | | 6 | 4.06 | 4.48 | 0.42 | 2 |
| Lanna, SE | | 6.1 | 558 | Clay soil | ND | 6.6 | Con | Spring barley | perennial ryegrass | NL | 100-110 | | 1 | 3.74 | 3.10 | -0.64 | 3 |
| Lanna, SE | | 6.1 | 558 | Clay soil | ND | 6.6 | Con | Spring Oats | perennial ryegrass | NL | 100-110 | | 1 | 5.00 | 4.40 | -0.60 | 3 |
| Mellby, SE | | 7.2 | 773 | Sandy loam soil | 1.58 | 5.9 | Con | Oat | perennial ryegrass | NL | 90 | | 1 | 3.10 | 2.70 | -0.40 | 4 |
| Mellby, SE | | 7.2 | 773 | Sandy loam soil | 1.58 | 5.9 | Con | Spring barley | perennial ryegrass | NL | 90 | | 1 | 5.10 | 4.80 | -0.30 | 4 |
| Mellby, SE | | 7.2 | 773 | Sandy loam soil | 1.58 | 5.9 | Con | Spring wheat | perennial ryegrass | NL | 110 | | 1 | 3.80 | 3.50 | -0.30 | 4 |
| South east Finland, FIN | | ND | 650 | Clay soil | ND | 6.3 | Con | Spring barley | Italian ryegrass | NL | 90 | | 5 | 4.70 | 4.65 | -0.05 | 5 |
| South east Finland, FIN | | ND | 651 | Silt soil | ND | 6.625.0 | Con | Spring barley | Italian ryegrass | NL | 90 | | 5 | 5.60 | 5.26 | -0.34 | 5 |
| South east Finland, FIN | | ND | 652 | Sandy soil | ND | 6.1 | Con | Spring barley | Italian ryegrass | NL | 90 | | 5 | 4.90 | 5.00 | 0.10 | 5 |
| South east Finland, FIN | | ND | 653 | Peat soil | ND | 5.075.0 | Con | Spring barley | Italian ryegrass | NL | 45 | | 5 | 5.90 | 4.87 | -1.03 | 5 |
| Jyndevad, DK | | 7.9 | 964 | Coarse sand | ND | 6.1 | Con | Spring barley | Mixtures of ryegrass and four clover species | M | 50-110 (manure) | | 12 | 1.85 | 2.27 | 0.42 | 6 |
| Foulum, DK | | 7.3 | 704 | Loamy sand | ND | 6.5 | Con | Spring barley | Mixtures of ryegrass and four clover species | M | 50-110 (manure) | | 12 | 3.00 | 3.63 | 0.63 | 6 |
| Flakkebjerg, DK | | 7.8 | 626 | Sandy loam | ND | 7.4 | Con | Spring barley | Mixtures of ryegrass and four clover species | M | 50-110 (manure) | | 12 | 2.13 | 2.68 | 0.55 | 6 |
| Canterbury, NZ | | 10 | 680 | Silt loam | ND | ND | Con | Winter wheat | Oats | NL | 0 | | 1 | 6.15 | 5.55 | -0.60 | 7 |
| Canterbury, NZ | | 10 | 680 | Silt loam | ND | ND | Con | Winter wheat | Winter wheat | NL | 0 | | 1 | 5.95 | 6.55 | 0.60 | 7 |
| Canterbury, NZ | | 10 | 680 | Silt loam | ND | ND | Con | Winter wheat | Oats | NL | 0 | | 2 | 6.50 | 4.70 | -1.80 | 7 |
| Canterbury, NZ | | 10 | 680 | Silt loam | ND | ND | Con | Winter wheat | Winter wheat | NL | 0 | | 2 | 6.00 | 5.40 | -0.60 | 7 |
| Canterbury, NZ | | 10 | 680 | Silt loam | ND | ND | Con | Winter wheat | Oats | NL | 50 | | 2 | 5.90 | 5.90 | 0.00 | 7 |
| Canterbury, NZ | | 10 | 680 | Silt loam | ND | ND | Con | Winter wheat | Winter wheat | NL | 50 | | 2 | 5.90 | 4.70 | -1.20 | 7 |
| Eastern Slovenia, SL | | 10.7 | 1047 | Loam/silt loam | 1.3-1.48 | 5.7-6.3 | Con | corn | Italian ryegrass | NL | 120 | | 3 | 9.70 | 9.50 | -0.20 | 8 |
| Eastern Slovenia, SL | | 10.7 | 1047 | Loam/silt loam | 1.3-1.48 | 5.7-6.3 | Con | corn | Winter rape | NL | 120 | | 3 | 9.70 | 9.70 | 0.00 | 8 |
| Eastern Slovenia, SL | | 10.7 | 1047 | Loam/silt loam | 1.3-1.48 | 5.7-6.3 | Con | corn | Subclover | L | 120 | | 3 | 9.70 | 10.60 | 0.90 | 8 |
| Eastern Slovenia, SL | | 10.7 | 1047 | Loam/silt loam | 1.3-1.48 | 5.7-6.3 | Con | corn | Crimson clover | L | 120 | | 3 | 9.70 | 10.60 | 0.90 | 8 |
| Georgetown, USA | | ND | 532 | Loamy sand | ND | ND | NT | Corn | Rye cover crops | NL | 224 + 280 (poultry manure) | | 3 | 7.90 | 8.00 | 0.10 | 9 |
| Georgetown, USA | | ND | 532 | Loamy sand | ND | ND | NT | Corn | Rye cover crops | NL | 224 + 280 (Compost poultry man) | | 3 | 7.40 | 7.90 | 0.50 | 9 |
| Georgetown, USA | | ND | 532 | Loamy sand | ND | ND | NT | Corn | Rye cover crops | NL | 224 | | 3 | 7.50 | 8.00 | 0.50 | 9 |
| Georgetown, USA | | ND | 532 | Loamy sand | ND | ND | Con | Corn | Rye cover crops | NL | 224 + 280 (poultry manure) | | 3 | 8.30 | 7.90 | -0.40 | 9 |
| Georgetown, USA | | ND | 532 | Loamy sand | ND | ND | Con | Corn | Rye cover crops | NL | 224 + 280 (Compost poultry manure) | | 3 | 8.10 | 7.90 | -0.20 | 9 |
| Georgetown, USA | | ND | 532 | Loamy sand | ND | ND | Con | Corn | Rye cover crops | NL | 224 | | 3 | 8.30 | 8.10 | -0.20 | 9 |
| Zaragoza, ES | | ND | 986 | Silt loam | ND | 8.2 | Con | corn | Barley | NL | 298 | | 2 | 16.65 | 13.85 | -2.80 | 10 |
| Zaragoza, ES | | ND | 986 | Silt loam | ND | 8.2 | Con | corn | Winter rape | NL | 281.5 | | 2 | 16.65 | 14.00 | -2.65 | 10 |
| Zaragoza, ES | | 14.7 | 322 | Silt loam | ND | 8.2 | Con | corn | Common vetch | L | 309 | | 2 | 16.65 | 17.80 | 1.15 | 10 |
| Jokioinen, FIN | | ND | ND | Clay and fine sand | ND | 6.26 | Con | Barley | White clover (low seed rate) | L | 85 | | 5 | 4.20 | 4.20 | 0.00 | 11 |
| Jokioinen, FIN | | ND | ND | Clay and fine sand | ND | 6.26 | Con | Barley | Red clover (low seed rate) | L | 85 | | 5 | 4.20 | 4.00 | -0.20 | 11 |
| Jokioinen, FIN | | ND | ND | Clay and fine sand | ND | 6.26 | Con | Barley | Black medic (low seed rate) | L | 85 | | 5 | 4.20 | 4.00 | -0.20 | 11 |
| Jokioinen, FIN | | ND | ND | Clay and fine sand | ND | 6.26 | Con | Barley | Westerworld grass (low seed rate) | NL | 85 | | 5 | 4.20 | 4.00 | -0.20 | 11 |
| Jokioinen, FIN | | ND | ND | Clay and fine sand | ND | 6.26 | Con | Barley | Timothy (low seed rate) | NL | 85 | | 5 | 4.20 | 4.20 | 0.00 | 11 |
| Jokioinen, FIN | | ND | ND | Clay and fine sand | ND | 6.26 | Con | Barley | Winter wheat (low seed rate) | NL | 85 | | 5 | 4.20 | 3.70 | -0.50 | 11 |
| Jokioinen, FIN | | ND | ND | Clay and fine sand | ND | 6.26 | Con | Barley | Italian ryegrass (low seed rate) | NL | 85 | | 5 | 4.20 | 4.00 | -0.20 | 11 |
| Jokioinen, FIN | | ND | ND | Clay and fine sand | ND | 6.26 | Con | Barley | White clover (standard seed rate) | L | 85 | | 5 | 4.30 | 4.10 | -0.20 | 11 |
| Jokioinen, FIN | | ND | ND | Clay and fine sand | ND | 6.26 | Con | Barley | Red clover (standard seed rate) | L | 85 | | 5 | 4.30 | 4.20 | -0.10 | 11 |
| Jokioinen, FIN | | ND | ND | Clay and fine sand | ND | 6.26 | Con | Barley | Black medic (standard seed rate) | L | 85 | | 5 | 4.30 | 4.10 | -0.20 | 11 |
| Jokioinen, FIN | | ND | ND | Clay and fine sand | ND | 6.26 | Con | Barley | Westerworld grass (standard seed rate) | NL | 85 | | 5 | 4.30 | 3.90 | -0.40 | 11 |
| Jokioinen, FIN | | ND | ND | Clay and fine sand | ND | 6.26 | Con | Barley | Timothy (standard seed rate) | NL | 85 | | 5 | 4.30 | 4.20 | -0.10 | 11 |
| Jokioinen, FIN | | ND | ND | Clay and fine sand | ND | 6.26 | Con | Barley | Winter wheat (standard seed rate) | NL | 85 | | 5 | 4.30 | 3.40 | -0.90 | 11 |
| Jokioinen, FIN | | ND | ND | Clay and fine sand | ND | 6.26 | Con | Barley | Italian ryegrass (standard seed rate) | NL | 85 | | 5 | 4.30 | 3.80 | -0.50 | 11 |
| Jokioinen, FIN | | ND | ND | Clay and fine sand | ND | 6.26 | Con | Barley | White clover (High seed rate) | L | 85 | | 5 | 4.10 | 3.80 | -0.30 | 11 |
| Jokioinen, FIN | | ND | ND | Clay and fine sand | ND | 6.26 | Con | Barley | Red clover (High seed rate) | L | 85 | | 5 | 4.10 | 3.90 | -0.20 | 11 |
| Jokioinen, FIN | | ND | ND | Clay and fine sand | ND | 6.26 | Con | Barley | Black medic (High seed rate) | L | 85 | | 5 | 4.10 | 4.10 | 0.00 | 11 |
| Jokioinen, FIN | | ND | ND | Clay and fine sand | ND | 6.26 | Con | Barley | Westerworld grass (High seed rate) | NL | 85 | | 5 | 4.10 | 3.60 | -0.50 | 11 |
| Jokioinen, FIN | | ND | ND | Clay and fine sand | ND | 6.26 | Con | Barley | Timothy (High seed rate) | NL | 85 | | 5 | 4.10 | 3.80 | -0.30 | 11 |
| Jokioinen, FIN | | ND | ND | Clay and fine sand | ND | 6.26 | Con | Barley | Winter wheat (High seed rate) | NL | 85 | | 5 | 4.10 | 2.80 | -1.30 | 11 |
| Jokioinen, FIN | | ND | ND | Clay and fine sand | ND | 6.26 | Con | Barley | Italian ryegrass (High seed rate) | NL | 85 | | 5 | 4.10 | 3.10 | -1.00 | 11 |
| Scotland, UK | | ND | 730 | Sandy loam/loamy sand | ND | ND | Con | Oat; crop rotation | Mustard | NL | ND | | 2 | 4.60 | 4.70 | 0.10 | 12 |
| Scotland, UK | | ND | 730 | Sandy loam/loamy sand | ND | ND | Con | Oat; crop rotation | Forage rape | NL | ND | | 2 | 4.60 | 4.60 | 0.00 | 12 |
| Scotland, UK | | ND | 730 | Sandy loam/loamy sand | ND | ND | Con | Oat; crop rotation | fodder radish | NL | ND | | 2 | 4.60 | 4.50 | -0.10 | 12 |
| Scotland, UK | | ND | 730 | Sandy loam/loamy sand | ND | ND | Con | Oat; crop rotation | Italian rye grass | NL | ND | | 2 | 4.60 | 3.90 | -0.70 | 12 |
| Scotland, UK | | ND | 730 | Sandy loam/loamy sand | ND | ND | Con | Oat; crop rotation | Winter peas | L | ND | | 2 | 4.60 | 4.20 | -0.40 | 12 |
| Scotland, UK | | ND | 730 | Sandy loam/loamy sand | ND | ND | Con | Oat; crop rotation | Grazing rye | NL | ND | | 2 | 4.60 | 4.20 | -0.40 | 12 |
| Scotland, UK | | ND | 730 | Sandy loam/loamy sand | ND | ND | Con | Oat; crop rotation | Winter wheat | NL | ND | | 2 | 4.60 | 4.40 | -0.20 | 12 |
| Scotland, UK | | ND | 730 | Sandy loam/loamy sand | ND | ND | Con | Oat; crop rotation | Winter barley | NL | ND | | 2 | 4.60 | 4.00 | -0.60 | 12 |
| South Jutland, DK | | 7.6 | 862 | Sandy loam | ND | ND | Con | Spring barley | Rye grass | NL | 110 (CAN) | | 3 | 5.30 | 5.10 | -0.20 | 13 |
| South Jutland, DK | | 7.6 | 862 | Sandy loam | ND | ND | Con | Spring barley | Rye grass | NL | 110 (slurry) | | 3 | 5.10 | 4.90 | -0.20 | 13 |
| South Jutland, DK | | 7.6 | 862 | Sandy loam | ND | ND | Con | Spring barley | Rye grass | NL | 165 (slurry) | | 3 | 5.20 | 5.30 | 0.10 | 13 |
| South Jutland, DK | | 7.6 | 862 | Sandy loam | ND | ND | Con | Spring barley | Rye grass | NL | 0 | | 2 | 2.00 | 1.80 | -0.20 | 13 |
| Hebei, CN | | 24.1 | 522 | Sandy loam | ND | 8.4 | Con | Spring corn | February Orchid | NL | 225 | | 5 | 3.20 | 9.70 | 6.50 | 14 |
| Marchfeld, AT | | 9.8 | 540 | Sandy loam to silty loam | ND | 7.6 | Con | Winter rye | Legume + non-legume | M | 0 | | 4 | 4.40 | 5.30 | 0.90 | 15 |
| Marchfeld, AT | | 9.8 | 540 | Sandy loam to silty loam | ND | 8.6 | Con | Winter rye | Legume | L | 0 | | 4 | 4.40 | 5.20 | 0.80 | 15 |
| Marchfeld, AT | | 9.8 | 540 | Sandy loam to silty loam | ND | 9.6 | Con | Winter rye | Non-legume | NL | 0 | | 4 | 4.40 | 5.60 | 1.20 | 15 |
| Marchfeld, AT | | 9.8 | 540 | Sandy loam to silty loam | ND | 7.6 | Con | Spring barley | Legume/non-legume | M | 0 | | 4 | 2.70 | 3.10 | 0.40 | 15 |
| Marchfeld, AT | | 9.8 | 540 | Sandy loam to silty loam | ND | 8.6 | Con | Spring barley | Legume | L | 0 | | 4 | 2.70 | 3.30 | 0.60 | 15 |
| Marchfeld, AT | | 9.8 | 540 | Sandy loam to silty loam | ND | 9.6 | Con | Spring barley | Non-legume | NL | 0 | | 4 | 2.70 | 2.70 | 0.00 | 15 |
| Jyndevad, DK | | ND | 859 | Coarse sandy | ND | 5.5-5.9 | Con | Spring barley | Grass | NL | 70 (manure) | | 3 | 2.80 | 3.30 | 0.50 | 16 |
| Jyndevad, DK | | ND | 859 | Coarse sandy | ND | 5.5-5.9 | Con | Spring barley | Clover | L | 0 | | 3 | 2.80 | 2.70 | -0.10 | 16 |
| Jyndevad, DK | | ND | 860 | Coarse sand | ND | ND | R | Spring barley | Rye grass | NL | 60-120 | | 4 | 4.20 | 4.78 | 0.58 | 17 |
| Jyndevad, DK | | ND | 860 | Coarse sand | ND | ND | R | Spring barley | Rye grass | NL | 60-120 | | 4 | 4.42 | 4.69 | 0.27 | 17 |
| 0dum, DK | | ND | 630 | Sandy loam | ND | ND | R | Spring barley | Rye grass | NL | 60-120 | | 4 | 4.57 | 4.65 | 0.08 | 17 |
| 0dum, DK | | ND | 630 | Sandy loam | ND | ND | R | Spring barley | Rye grass | NL | 60-120 | | 4 | 4.61 | 4.59 | -0.02 | 17 |
| 0dum, DK | | ND | 630 | Sandy loam | ND | ND | R | Spring barley | Rye grass | NL | 60-120 | | 4 | 4.57 | 4.65 | 0.08 | 17 |
| 0dum, DK | | ND | 630 | Sandy loam | ND | ND | R | Spring barley | Rye grass | NL | 60-120 | | 4 | 4.61 | 4.59 | -0.02 | 17 |
| England, UK | | ND | 113-241 | Multi soil types | ND | 5.3-8.1 | MT | Spring barley | Volunteer crop | M | 0-180 | | 3 | 4.95 | 4.79 | -0.16 | 18 |
| England, UK | | ND | 113-241 | Multi soil types | ND | 5.3-8.1 | MT | Spring barley | Phacelia | NL | 0-180 | | 3 | 4.95 | 4.96 | 0.01 | 18 |
| England, UK | | ND | 113-241 | Multi soil types | ND | 5.3-8.1 | MT | Spring barley | Ryegrass | NL | 0-180 | | 3 | 4.95 | 4.81 | -0.14 | 18 |
| England, UK | | ND | 113-241 | Multi soil types | ND | 5.3-8.1 | MT | Spring barley | Mustard | NL | 0-180 | | 3 | 4.95 | 4.95 | 0.00 | 18 |
| England, UK | | ND | 113-241 | Multi soil types | ND | 5.3-8.1 | MT | Spring barley | Forage rape | NL | 0-180 | | 3 | 4.95 | 4.87 | -0.08 | 18 |
| England, UK | | ND | 113-241 | Multi soil types | ND | 5.3-8.1 | MT | Spring barley | Forage rye | NL | 0-180 | | 3 | 4.95 | 4.74 | -0.21 | 18 |
| Poznan, PL | | 11.4 | 277 | Sandy loam | ND | 6.5 | MT | Spring barley | Straw | NL | 0-100 | | 3 | 3.77 | 3.49 | -0.28 | 19 |
| Poznan, PL | | 11.4 | 277 | Sandy loam | ND | 6.5 | MT | Spring barley | White mustard | NL | 0-100 | | 3 | 3.77 | 4.27 | 0.50 | 19 |
| Poznan, PL | | 11.4 | 277 | Sandy loam | ND | 6.5 | MT | Spring barley | Oats+ pea | M | 0-100 | | 3 | 3.77 | 4.94 | 1.17 | 19 |
| Poznan, PL | | 11.4 | 277 | Sandy loam | ND | 6.5 | MT | Spring barley | Phacelia | NL | 0-100 | | 3 | 3.77 | 4.16 | 0.39 | 19 |
| Canterbury, NZ | | 11.4 | 640 | ND | 1.5 | ND | ND | Silage corn | Forage wheat | NL | 175 | | 3 | ND | ND | ND | 20 |
| Hunan, CN | | 17.1 | 1500 | ND | ND | 5.42 | ND | Late rice | Ryegrass | NL | 224 | | 4 | 6.74 | 7.39 | 0.65 | 21 |
| Hunan, CN | | 17.1 | 1500 | ND | ND | 5.4 | ND | Late rice | Milk vetch | L | 224 | | 4 | 6.74 | 7.30 | 0.56 | 21 |
| Hunan, CN | | 17.1 | 1500 | ND | ND | 5.34 | ND | Late rice | Rape | NL | 224 | | 4 | 6.74 | 7.17 | 0.44 | 21 |
| Hunan, CN | | 17.1 | 1500 | ND | ND | 5.47 | ND | Late rice | Potato | NL | 224 | | 4 | 6.74 | 7.52 | 0.78 | 21 |
| Hunan, CN | | 17.1 | 1500 | ND | ND | 4.5-5.8 | ND | Early rice | Ryegrass | NL | 175 | | 4 | 6.09 | 5.36 | -0.73 | 22 |
| Hunan, CN | | 17.1 | 1500 | ND | ND | 4.5-5.9 | ND | Early rice | Milk vetch | L | 175 | | 4 | 6.09 | 5.64 | -0.45 | 22 |
| Hunan, CN | | 17.1 | 1500 | ND | ND | 4.5-5.10 | ND | Early rice | Rape | NL | 175 | | 4 | 6.09 | 5.32 | -0.78 | 22 |
| Guizhou, CN | | 11.2 | 739 | ND | 1.2 | ND | ND | corn | Potato | NL | ND | | 1 | 6.09 | 6.57 | 0.47 | 23 |
| Hubei, CN | | 18.3 | 1313 | ND | ND | 6.4 | ND | Early rice | Milk vetch | L | 157.2 | | 1 | 6.70 | 7.18 | 0.48 | 24 |
| Hubei, CN | | 16.7 | 1450 | ND | ND | ND | ND | Early rice | Milk vetch | L | 123.8 | | 2 | 5.72 | 6.21 | 0.49 | 24 |
| Jiangsu, CN | | 15.5 | 1038 | ND | ND | 7.6 | ND | Rice | Milk vetch | L | 240 | | 1 | 7.42 | 9.30 | 1.88 | 25 |
| Jiangsu, CN | | 15.7 | 1177 | ND | ND | 6.23 | ND | Rice | Milk vetch | L | 270 | | 1 | 6.32 | 6.67 | 0.34 | 26 |
| Jiangsu, CN | | 15.7 | 1177 | ND | ND | 6.23 | ND | Rice | Ryegrass | NL | 270 | | 1 | 6.32 | 6.50 | 0.18 | 26 |
| Henan, CN | | 15.2 | 1150 | ND | ND | 6.7 | ND | Rice | Milk vetch | L | 217.5 | | 1 | 7.99 | 9.29 | 1.30 | 27 |
| Zhejiang, CN | | 18.3 | 2000 | ND | ND | 5.3 | ND | Rice | Milk vetch | L | 181.3 | | 2 | 7.52 | 9.24 | 1.73 | 28 |
| Henan, CN | | 15.2 | 1150 | ND | ND | ND | ND | Rice | Milk vetch | L | 27.2 (organic） | | 1 | 7.76 | 9.20 | 1.44 | 29 |
| Ontario, CA | | 10.3 | 758 | Sandy loam | ND | 7.4 | Con | Corn | Alfalfa | L | 0 | | 1 | 11.9 | 12.4 | 0.5 | 30 |
| Ontario, CA | | 10.3 | 758 | Sandy loam | ND | 7.4 | Con | Corn | Crimson clover | L | 0 | | 1 | 11.9 | 10.1 | -1.8 | 30 |
| Ontario, CA | | 10.3 | 758 | Sandy loam | ND | 7.4 | Con | Corn | Red clover | L | 0 | | 1 | 11.9 | 11.1 | -0.8 | 30 |
| Ontario, CA | | 10.3 | 758 | Sandy loam | ND | 7.4 | Con | Corn | Alfalfa | L | 112 | | 1 | 12.8 | 12.4 | -0.4 | 30 |
| Ontario, CA | | 10.3 | 758 | Sandy loam | ND | 7.4 | Con | Corn | Crimson clover | L | 112 | | 1 | 12.8 | 10.1 | -2.7 | 30 |
| Ontario, CA | | 10.3 | 758 | Sandy loam | ND | 7.4 | Con | Corn | Red clover | L | 112 | | 1 | 12.8 | 11.1 | -1.7 | 30 |
| Ontario, CA | | 10.3 | 758 | Sandy loam | ND | 7.4 | Con | Corn | Alfalfa | L | 224 | | 1 | 13.8 | 12.4 | -1.4 | 30 |
| Ontario, CA | | 10.3 | 758 | Sandy loam | ND | 7.4 | Con | Corn | Crimson clover | L | 224 | | 1 | 13.8 | 10.1 | -3.6 | 30 |
| Ontario, CA | | 10.3 | 758 | Sandy loam | ND | 7.4 | Con | Corn | Red clover | L | 224 | | 1 | 13.8 | 11.1 | -2.7 | 30 |
| Ontario, CA | | 10.3 | 758 | Loam | ND | 7.4 | Con | Corn | Alfalfa | L | 0 | | 1 | 8.9 | 6.8 | -2.1 | 30 |
| Ontario, CA | | 10.3 | 758 | Loam | ND | 7.4 | Con | Corn | Crimson clover | L | 0 | | 1 | 8.9 | 6.4 | -2.5 | 30 |
| Ontario, CA | | 10.3 | 758 | Loam | ND | 7.4 | Con | Corn | Red clover | L | 0 | | 1 | 8.9 | 7.5 | -1.4 | 30 |
| Ontario, CA | | 10.3 | 758 | Loam | ND | 7.4 | Con | Corn | Alfalfa | L | 112 | | 1 | 11.3 | 6.8 | -4.5 | 30 |
| Ontario, CA | | 10.3 | 758 | Loam | ND | 7.4 | Con | Corn | Crimson clover | L | 112 | | 1 | 11.3 | 6.4 | -4.9 | 30 |
| Ontario, CA | | 10.3 | 758 | Loam | ND | 7.4 | Con | Corn | Red clover | L | 112 | | 1 | 11.3 | 7.5 | -3.8 | 30 |
| Ontario, CA | | 10.3 | 758 | Loam | ND | 7.4 | Con | Corn | Alfalfa | L | 224 | | 1 | 10.1 | 6.8 | -3.3 | 30 |
| Ontario, CA | | 10.3 | 758 | Loam | ND | 7.4 | Con | Corn | Crimson clover | L | 224 | | 1 | 10.1 | 6.4 | -3.7 | 30 |
| Ontario, CA | | 10.3 | 758 | Loam | ND | 7.4 | Con | Corn | Red clover | L | 224 | | 1 | 10.1 | 7.5 | -2.6 | 30 |
| Hunan, CN | | 17.5 | 1350 | Silt loam | ND | 6.0 | Con | Early rice, late rice | Ryegrass | NL | 0 | | 3 | 9.8 | 6.3 | -3.5 | 31 |
| Hunan, CN | | 17.5 | 1350 | Silt loam | ND | 6.0 | Con | Early rice, late rice | Ryegrass | NL | 200 | | 3 | 11.7 | 10.6 | -1.1 | 31 |
| Hunan, CN | | 17.5 | 1350 | Silt loam | ND | 6.0 | Con | Early rice, late rice | Ryegrass | NL | 400 | | 3 | 12.9 | 11.9 | -1.0 | 31 |
| Shannxi, CN | | 11.0 | 582 | Silt loam | 1.21 | ND | Con | Wheat | Legume | L | 180 | | 3 | 4.2 | 3.9 | -0.3 | 32 |
| Shannxi, CN | | 11.0 | 582 | Silt loam | 1.26 | ND | Con | Wheat | Legume | L | 180 | | 3 | 1.9 | 1.8 | -0.1 | 32 |
| Shannxi, CN | | 11.0 | 582 | Silt loam | 1.29 | ND | Con | Wheat | Legume | L | 180 | | 3 | 3.9 | 4.0 | 0.1 | 32 |
| Beijing, CN | | ND | ND | Silt loam | 1.42 | 6.1 | Con | Cucumber | Sweet corn | NL | 1222(org) (2 year total) | | 2 | 255.0 | 243.0 | -12.0 | 33 |
| Illinois, USA | | 11.3 | 1015 | Silt | 1.57 | 6.7 | Con | Chickpea | Forage radish | NL | Manure | | 1 | 2.2 | 2.2 | 0.0 | 34 |
| Illinois, USA | | 11.3 | 1015 | Silt | 1.57 | 6.7 | Con | Chickpea | Mixture of forage radish a buckwheat | NL | Manure | | 1 | 2.2 | 2.1 | -0.1 | 34 |
| Illinois, USA | | 11.3 | 1015 | Silt | 1.57 | 6.7 | Con | Chickpea | FRhvr | NL | Manure | | 1 | 2.2 | 2.2 | 0.0 | 34 |
| Illinois, USA | | 11.3 | 1015 | Silt | 1.55 | 6.3 | Con | Corn | Forage radish | NL | Manure | | 1 | 2.5 | 2.4 | -0.2 | 34 |
| Illinois, USA | | 11.3 | 1015 | Silt | 1.55 | 6.3 | Con | Corn | Mixture of forage radish a buckwheat | NL | Manure | | 1 | 2.5 | 2.5 | 0.0 | 34 |
| Illinois, USA | | 11.3 | 1015 | Silt | 1.55 | 6.3 | Con | Corn | FRhvr | NL | Manure | | 1 | 2.5 | 2.0 | -0.5 | 34 |
| Ningxia, CN | | ND | ND | ND | 1.37-1.60 | 7.88 | Con | Tomato | Feed corn | NL |  | | 1 | 81.5 | 91.7 | 10.2 | 35 |
| Ningxia, CN | | ND | ND | ND | 1.37-1.60 | 7.88 | Con | Tomato | Sweet corn | NL |  | | 1 | 81.5 | 96.6 | 15.1 | 35 |
| Beijing, CN | | ND | ND | ND | ND | 8.0-8.3 | Con | Spinach | Sweet corn | NL | 310 (high irrigation) | | 4 | 21.9 | 18.4 | -3.5 | 36 |
| Beijing, CN | | ND | ND | ND | ND | 8.0-8.3 | Con | Spinach | Sweet corn | NL | 310 | | 4 | 22.0 | 20.4 | -1.6 | 36 |
| Beijing, CN | | ND | ND | ND | ND | 8.0-8.3 | Con | Spinach | Sweet corn | NL | 220 (high irrigation) | | 4 | 20.2 | 20.3 | 0.1 | 36 |
| Beijing, CN | | ND | ND | ND | ND | 8.0-8.3 | Con | Spinach | Sweet corn | NL | 233 | | 4 | 18.7 | 20.7 | 2.0 | 36 |
| Hebei, CN | | 13 | 523 | Sandy loam | 1.37 | 7.50 | Con | Cucumber | Sweet corn | NL | 728 (U) + 430 (org) | | 3 | 271.0 | 259.0 | -12.0 | 37 |
| Hebei, CN | | 13 | 523 | Sandy loam | 1.37 | 7.50 | Con | Cucumber | Amaranth | NL | 728 (U) + 430 (org) | | 3 | 271.0 | 255.0 | -16.0 | 37 |
| Hebei, CN | | 13 | 523 | Sandy loam | 1.37 | 7.50 | Con | Cucumber | Sweet sorghum | NL | 728 (U) + 430 (org) | | 3 | 271.0 | 253.0 | -18.0 | 37 |
| Beijing, CN | | ND | ND | ND | ND | 7.8 | Con | Fennel | Sweet corn | NL | 360 (U) + 75 (org) | | 1 | 0.0 | 0.0 | 0.0 | 38 |
| Canterbury, NZ | | 10 | 680 | Silt loam | ND | ND | Con | Winter wheat | Oats | NL | 0 | | 1 | 6.2 | 5.6 | -0.6 | 39 |
| Canterbury, NZ | | 10 | 680 | Silt loam | ND | ND | Con | Winter wheat | Winter wheat | NL | 0 | | 1 | 6.0 | 6.6 | 0.6 | 39 |
| Canterbury, NZ | | 10 | 680 | Silt loam | ND | ND | Con | Winter wheat | Oats | NL | 0 | | 2 | 6.5 | 4.7 | -1.8 | 39 |
| Canterbury, NZ | | 10 | 680 | Silt loam | ND | ND | Con | Winter wheat | Winter wheat | NL | 0 | | 2 | 6.0 | 5.4 | -0.6 | 39 |
| Canterbury, NZ | | 10 | 680 | Silt loam | ND | ND | Con | Winter wheat | Oats | NL | 50 | | 2 | 5.9 | 5.9 | 0.0 | 39 |
| Canterbury, NZ | | 10 | 680 | Silt loam | ND | ND | Con | Winter wheat | Winter wheat | NL | 50 | | 2 | 5.9 | 4.7 | -1.2 | 39 |
| Jyevad, DK | | 7.9 | 859 | Loamy sand | ND | ND | Con | Spring barley, spring wheat | perennial ryegrass, Italian ryegrass | NL | 135 | | 28 | ND | ND | 13.0 | 40 |
|  | |  |  |  |  |  |  |  |  |  |  | |  |  |  |  |  |

MAAT - mean annual air temperature (^o^C) and MAP - mean annual precipitation. Grain yield under control (C), grain yield under cover crops (CC) and changes in grain yield (∆G) were calculated in t ha^-1^. ^a^Different methods were used to measure soil pH using pH probe/ meter in deionized water or 0.01 M CaCl_2_ in 1:1 and 1:2, or 1:5 (v: v) soils: solution ratios. ND= no data available; L= legume; NL= non-legume and M= mixed. Org= organic; U= urine; FRhvr= mix of forage radish, hairy vetch, and cereal rye. Con= conventional; R= reduced; NT= no-till; MT= Multi-tillage. AT= Austria; CA= Canada; DK= Denmark; CN= China; SE= Sweden; NZ= New Zealand; USA= United States of America; ES= Spain; FIN= Finland; PL= Poland; UK= United Kingdom. Ref.:1= Torstensson &Aronsson (2000); 2= Torstensson et al. (2006); 3= Aronsson et al. (2011); 4= Stenberg et al. (1999); 5= Lemola and Turtola (2000); 6= Doltra and Olesen (2013); 7= Francis (1995); 8= Kramberger et al. (2009);9= Ritter et al. (1998); 10= Salmerón et al. (2010); 11= Kankanen and Eriksson (2007); 12= Baggs et al. (2000); 13= Thomsen (2005); 14= Bai et al. (2015); 15= Rinnofner et al. (2008); 16= Askegaard and Eriksen (2008); 17= Hansen and Djurhuus (1997); 18= Richards et al. (1996); 19=Małecka & Blecharczyk (2008); 20= Teixeira et al. (2016); 21= Tang et al.(2010); 22= Wang et al.(2006); 23= Zhang et al. (2006); 24= Li et al. (2012); 25= Hu et al. (2013); 26= Qiao et al. (2011); 27= Lu et al. (2013); 28= Zhu et al. (2011); 29= Liu et al. (2012); 30= Coombs et al.(2017); 31= Zhu et al.(2016); 32= Zhang et al.(2009); 33= Guo et al.(2008); 34= Welch et al.(2016); 35= Mao et al.(2015); 36= Ren et al.(2006); 37= Peng et al. (2015); 38= Xi et al. (2011); 39= Francis (1995); 40= Berntsen et al. (2006).
