# Supplementary material for: A critical review of the impacts of cover crops on nitrogen leaching, net greenhouse gas balance and crop productivity
Source: Glob Chang Biol. 2019 May 13;25(8):2530–43. doi: 10.1111/gcb.14644 (PMC6851768; doi:10.1111/gcb.14644)
Supplement: Supplementary file 3 [file GCB-25-2530-s003.docx]

Table 3: Published studies on the impacts of cover crops, climate and soil properties on N in the grain of primary crop.

| Location  (country/state) | MAAT  (^o^C) | | MAP  (mm) | Soil  texture | BD  (g cm^-3^) | | pH^a^ | Tillage | Primary crop (C ) | Cover crops (CC) | Type of CC | Added N (kg ha^-1^) | Duration  (year) | | Grain-N content under C (g m^-2^) | Grain-N content under CC (g m^-2^) | ∆N (g m^-2^) | Ref. |
| --- | --- | --- | --- | --- | --- | --- | --- | --- | --- | --- | --- | --- | --- | --- | --- | --- | --- | --- |
| Mellby, SE | | 7.2 | 803 | Sandy loam soil | | ND | ND | Con | Cereals; potatoes | perennial ryegrass | NL | 97 | | 5 | 6.7 | 6.3 | -0.4 | 1 |
| Mellby, SE | | 7.2 | 803 | Sandy loam soil | | ND | ND | Con | Cereals; potatoes | perennial ryegrass | NL | 161 | | 6 | 7.5 | 7.2 | -0.3 | 1 |
| Mellby, SE | | 7.2 | 803 | Sandy loam soil | | ND | ND | Con | Cereals; potatoes | perennial ryegrass | NL | 263 | | 7 | 8.3 | 8.2 | -0.1 | 1 |
| Lilla Boslid, SE | | 7.2 | 803 | Sandy soil | | ND | 6.1 | Con | Spring barley | perennial ryegrass | NL | 90 | | 2 | 6.8 | 7.5 | 0.7 | 2 |
| Lanna, SE | | 6.1 | 558 | Clay soil | | ND | 6.6 | Con | Spring barley | perennial ryegrass | NL | 105 | | 2 | 7.1 | 6 | -1.1 | 2 |
| Lanna, SE | | 6.1 | 558 | Clay soil | | ND | 6.6 | Con | Spring Oats | perennial ryegrass | NL | 105 | | 2 | 8.1 | 6.7 | -1.4 | 2 |
| Mellby, SE | | 7.2 | 773 | Sandy loam soil | | 1.58 | 5.9 | Con | Oat | perennial ryegrass | NL | 90 | | 1 | 6.9 | 6 | -0.9 | 3 |
| Mellby, SE | | 7.2 | 773 | Sandy loam soil | | 1.58 | 5.9 | Con | Spring barley | perennial ryegrass | NL | 90 | | 1 | 6.9 | 6 | -0.9 | 3 |
| Mellby, SE | | 7.2 | 773 | Sandy loam soil | | 1.58 | 5.9 | Con | Spring wheat | perennial ryegrass | NL | 110 | | 1 | 6.9 | 6 | -0.9 | 3 |
| South east Finland, FIN | | ND | 650 | Clay soil | | ND | 6.3 | Con | Spring barley | Italian ryegrass | NL | 90 | | 5 | 7.5 | 7.1 | -0.4 | 4 |
| South east Finland, FIN | | ND | 651 | Silt soil | | ND | 6.6 | Con | Spring barley | Italian ryegrass | NL | 90 | | 5 | 7.9 | 7.7 | -0.2 | 4 |
| South east Finland, FIN | | ND | 652 | Sandy soil | | ND | 6.1 | Con | Spring barley | Italian ryegrass | NL | 90 | | 5 | 7.8 | 7.4 | -0.4 | 4 |
| South east Finland, FIN | | ND | 653 | Peat soil | | ND | 5.1 | Con | Spring barley | Italian ryegrass | NL | 45 | | 5 | 10.6 | 9.7 | -0.9 | 4 |
| Jyndevad, DK | | 7.9 | 964 | Coarse sand | | ND | 6.1 | Con | Spring barley | Mixtures of ryegrass and four clover species | M | 80 | | 12 | 2.6 | 3 | 0.4 | 5 |
| Foulum, DK | | 7.3 | 704 | Loamy sand | | ND | 6.5 | Con | Spring barley | Mixtures of ryegrass and four clover species | M | 80 | | 12 | 4.2 | 5.6 | 1.4 | 5 |
| Flakkebjerg, DK | | 7.8 | 626 | Sandy loam | | ND | 7.4 | Con | Spring barley | Mixtures of ryegrass and four clover species | M | 80 | | 12 | 3 | 3.8 | 0.8 | 5 |
| Canterbury, NZ | | 11.4 | 680 | Silt loam | | 1.4 | 5.4 | Con | Cereals (barley; wheat) | Forage rape | NL | 63 | | 7 | 15 | 15.8 | 0.8 | 6 |
| Canterbury, NZ | | 11.4 | 680 | Silt loam | | 1.4 | 5.4 | Con | Cereals (barley; wheat) | Forage rape | NL | 63 | | 7 | 15.3 | 15.7 | 0.4 | 6 |
| Canterbury, NZ | | 11.4 | 680 | Silt loam | 1.4 | | 5.4 | Con | Cereals (barley; wheat) | Forage rape | NL | 63 | | 7 | 15.5 | 15.9 | 0.4 | 6 |
| Canterbury, NZ | | 11.4 | 680 | Silt loam | 1.4 | | 5.4 | Con | Peas | Forage rape | NL | 63 | | 8 | 21 | 19.4 | -1.6 | 6 |
| Canterbury, NZ | | 11.4 | 680 | Silt loam | 1.4 | | 5.4 | Con | Peas | Forage rape | NL | 63 | | 9 | 21.2 | 20.4 | -0.8 | 6 |
| Canterbury, NZ | | 11.4 | 680 | Silt loam | 1.4 | | 5.4 | Con | Peas | Forage rape | NL | 63 | | 10 | 18.6 | 19.2 | 0.6 | 6 |
| Eastern Slovenia, SL | | 10.7 | 1047 | Loam/silt loam | 1.3-1.48 | | 5.7-6.3 | Con | corn | Italian ryegrass | NL | 120 | | 3 | 12.5 | 12.1 | -0.4 | 7 |
| Eastern Slovenia, SL | | 10.7 | 1047 | Loam/silt loam | 1.3-1.48 | | 5.7-6.3 | Con | corn | Winter rape | NL | 120 | | 3 | 12.5 | 13.4 | 0.9 | 7 |
| Eastern Slovenia, SL | | 10.7 | 1047 | Loam/silt loam | 1.3-1.48 | | 5.7-6.3 | Con | corn | Sub-clover | L | 120 | | 3 | 12.5 | 15.3 | 2.8 | 7 |
| Eastern Slovenia, SL | | 10.7 | 1047 | Loam/silt loam | 1.3-1.48 | | 5.7-6.3 | Con | corn | Crimson clover | L | 120 | | 3 | 12.5 | 15.5 | 3.0 | 7 |
| Jutland, DK | | 7.6 | 862 | Sandy loam | ND | | ND | Con | Spring barley | Rye grass | NL | 110 | | 3 | 6.6 | 6.5 | -0.1 | 8 |
| South Jutland, DK | | 7.6 | 862 | Sandy loam | ND | | ND | Con | Spring barley | Rye grass | NL | 110 | | 3 | 8 | 6.8 | -1.2 | 8 |
| Jutland, DK | | 7.6 | 862 | Sandy loam | ND | | ND | Con | Spring barley | Rye grass | NL | 165 | | 3 | 8.8 | 10.1 | 1.3 | 8 |
| Jutland, DK | | 7.6 | 862 | Sandy loam | ND | | ND | Con | Spring barley | Rye grass | NL | 0 | | 3 | 29 | 22 | -7.0 | 8 |
| Marchfeld, AT | | 9.8 | 540 | Sandy loam to silty loam | ND | | 9.6 | Con | Winter rye | Non-legume + legume | M | 0 | | 4 | 7.4 | 8.6 | 1.2 | 9 |
| Marchfeld, AT | | 9.8 | 540 | Sandy loam to silty loam | ND | | 9.6 | Con | Winter rye | legume | L | 0 | | 4 | 7.4 | 8.7 | 1.3 | 9 |
| Marchfeld, AT | | 9.8 | 540 | Sandy loam to silty loam | ND | | 9.6 | Con | Winter rye | Non-legume | L | 0 | | 4 | 7.4 | 10.2 | 2.8 | 9 |
| Marchfeld, AT | | 9.8 | 540 | Sandy loam to silty loam | ND | | 9.6 | Con | Spring barley | Non-legume+ legume | M | 0 | | 4 | 4.5 | 4.6 | 0.1 | 9 |
| Marchfeld, AT | | 9.8 | 540 | Sandy loam to silty loam | ND | | 9.6 | Con | Spring barley | Legume | L | 0 | | 4 | 4.5 | 5.7 | 1.2 | 9 |
| Marchfeld, AT | | 9.8 | 540 | Sandy loam to silty loam | ND | | 9.6 | Con | Spring barley | Non-legume | NL | 0 | | 4 | 4.5 | 4 | -0.5 | 9 |
| Jyndevad, DK | | ND | 859 | Coarse sandy | ND | | 5.5-5.9 | Con | Spring barley | Grass | NL | 70 | | 3 | 3.4 | 4.3 | 0.9 | 10 |
| Jyndevad, DK | | ND | 859 | Coarse sandy | ND | | 5.5-5.9 | Con | Spring barley | Clover | L | 0 | | 3 | 3.4 | 3.5 | 0.1 | 10 |
| Poznan, PL | | 11.4 | 277 | Sandy loam | ND | | 6.5 | MT | Spring barley | Straw | NL | 50 | | 3 | 5.6 | 5.3 | -0.3 | 11 |
| Poznan, PL | | 11.4 | 277 | Sandy loam | ND | | 6.5 | MT | Spring barley | White mustard | NL | 50 | | 3 | 5.6 | 6.3 | 0.7 | 11 |
| Poznan, PL | | 11.4 | 277 | Sandy loam | ND | | 6.5 | MT | Spring barley | Oats+ pea | M | 50 | | 3 | 5.6 | 7.9 | 2.3 | 11 |
| Poznan, PL | | 11.4 | 277 | Sandy loam | ND | | 6.5 | MT | Spring barley | Phacelia | L | 50 | | 3 | 5.6 | 6.4 | 0.8 | 11 |
| Jiangsu, CN | | 15.5 | 1038 | ND | ND | | 7.6 | ND | Rice | Milk vetch | L | 0 | | 1 | 9.2 | 11.6 | 2.4 | 12 |
| Zhejiang, CN | | 17.9 | 1500 | ND | ND | | 5.4 | ND | Rice | Milk vetch | L | 0 | | 2 | 8.8 | 11.3 | 0.5 | 13 |
| Fujian, CN | | 17.0 | 1674 | ND | 1.4 | | 4.8 | ND | Rice | Milk vetch | L | 0 | | 4 | 9.1 | 9.6 | 2.5 | 13 |
| Zhejiang, CN | | 17.9 | 1500 | ND | ND | | 5.4 | ND | Rice | Milk vetch | L | 0 | | 2 | 8.9 | 10.6 | 1.6 | 13 |
| Northwest of Ames, USA | | 9.3 | 856 | Fine-loamy | ND | | ND | NT | Soybean | Oats | NL | 0 | | 3 | 3.1 | 2.6 | -0.5 | 14 |
| Northwest of Ames, USA | | 9.3 | 856 | Fine-loamy | ND | | ND | NT | Soybean | Rye | NL | 0 | | 3 | 3.1 | 2.9 | -0.2 | 14 |
| Northwest of Ames, USA | | 9.3 | 856 | Fine-loamy | ND | | ND | NT | Corn | Oats | NL | 207 | | 3 | 12.6 | 12.0 | -0.6 | 14 |
| Northwest of Ames, USA | | 9.3 | 856 | Fine-loamy | ND | | ND | NT | Corn | Rye | NL | 207 | | 3 | 12.6 | 12.6 | 0.0 | 14 |
| Beijing, CN | | ND | ND | ND | ND | | 8.0-8.3 | Con | Spinach | Sweet corn | NL | 310(high irrigation) | | 4 | 11.57 | 10.40 | -1.2 | 15 |
| Beijing, CN | | ND | ND | ND | ND | | 8.0-8.3 | Con | Spinach | Sweet corn | NL | 310 | | 4 | 12.42 | 11.29 | -1.1 | 15 |
| Beijing, CN | | ND | ND | ND | ND | | 8.0-8.3 | Con | Spinach | Sweet corn | NL | 220 (high irrigation) | | 4 | 10.41 | 10.77 | 0.4 | 15 |
| Beijing, CN | | ND | ND | ND | ND | | 8.0-8.3 | Con | Spinach | Sweet corn | NL | 233 | | 4 | 10.13 | 11.69 | 1.6 | 15 |
| Hebei, CN | | 13.0 | 523 | Say loam | 1.37 | | 7.5 | Con | Cucumber | Sweet corn | NL | 728 (U) + 430 (org) | | 3 | 104.8 | 84.5 | -20.3 | 16 |
| Hebei, CN | | 13.0 | 523 | Say loam | 1.37 | | 7.5 | Con | Cucumber | Amaranth | NL | 728 (U) +430 (org) | | 3 | 104.8 | 88.8 | -16.0 | 16 |
| Hebei, CN | | 13.0 | 523 | Say loam | 1.37 | | 7.5 | Con | Cucumber | Sweet sorghum | NL | 728 (U) + 430 (org) | | 3 | 104.8 | 77.5 | -27.3 | 16 |
| Beijing, CN | | ND | ND | ND | ND | | 7.8 | Con | Fennel | Sweet corn | NL | 360 (U) +75 (org) | | 1 | 103.7 | 107.1 | 3.4 | 17 |

MAAT - mean annual air temperature (^o^C) and MAP - mean annual precipitation. ^a^Different methods were used to measure soil pH using pH probe/ meter in deionized water or 0.01 M CaCl_2_ in 1:1 and 1:2, or 1:5 (v: v) soils: solution ratios. ND= no data available; L= legume; NL= non-legume and M= mixed. Org= organic; U= urine; Con= conventional; NT= no-till. AT= Austria; DK= Denmark; CN= China; SL= Slovenia; NZ= New Zealand; USA= United States of America; SE= Sweden; FIN= Finland; PL= Poland. Ref.:1= Torstensson &Aronsson (2000); 2= Aronsson et al. (2011); 3= Stenberg et al. (1999); 4= Lemola and Turtola (2000); 5= Doltra and Olesen (2013); 6= Fraser et al. (2013); 7= Kramberger et al. (2009); 8= Thomsen (2005); 9= Rinnofner et al. (2008); 10= Askegaard and Eriksen (2008); 11= Małecka & Blecharczyk (2008); 12= Hu et al. (2013); 13= Wang et al.(2014);14= Kaspar et al. (2012); 15= Ren et al.(2006); 16= Peng et al. (2015); 17= Xi et al. (2011).
