# Supplementary material for: A critical review of the impacts of cover crops on nitrogen leaching, net greenhouse gas balance and crop productivity
Source: Glob Chang Biol. 2019 May 13;25(8):2530–43. doi: 10.1111/gcb.14644 (PMC6851768; doi:10.1111/gcb.14644)
Supplement: Supplementary file 4 [file GCB-25-2530-s004.docx]

Table 4: Published studies on the impacts of cover crops, climate and soil properties on soil organic carbon (SOC) at the top 30 cm soil depth.

| Location  (country/state) | MAAT  (^o^C) | MAP  (mm) | Climate zone | Soil  texture | BD  (g cm^-3^) | pH^a^ | Tillage | Primary crop (C) | Cover crops (CC) | Type of CC | Added N (kg ha^-1^) | Duration  (year) | Original depth  (cm) | SOC under C (0-30cm; t C ha^-1^)¶ | SOC under CC (0-30cm; t C ha^-1^)¶ | | ∆ SOC at 0-30cm; t ha^-1^)** | Ref. |
| --- | --- | --- | --- | --- | --- | --- | --- | --- | --- | --- | --- | --- | --- | --- | --- | --- | --- | --- |
| Boigneville, FR | 11.5 | 604 | MC | Loamy sand/coarse | 1.42 | ND | Con | Winter wheat | White mustard | NL | 103 | 16 | 30 | 44.7 | 45.7 | 1.00 | | 1 |
|  | 11.5 | 604 | MC | Loamy sand/coarse | 1.52 | ND | NT | Winter wheat | White mustard | NL | 103 | 16 | 28 | 44.2 | 45.1 | 0.90 | | 1 |
| Kerlavic, FR | 12.1 | 1213 | MC | Loamy sand/sandy | 1.26 | ND | Con | Winter wheat | Italian ryegrass | NL | 125 | 13 | 30 | 98.6 | 102.9 | 4.30 | | 1 |
| Thibie, FR | 10.8 | 605 | MC | Loam | 1.36 | ND | Con | Winter wheat | Radish/winter cereal | NL | 84 | 13 | 30 | 59.7 | 61.7 | 2.00 | | 1 |
| Thibie, FR | 10.8 | 605 | MC | Loam | 1.40 | ND | Con | Winter wheat | Radish/winter cereal | NL | 123 | 17 | 23.5 | 50.5 | 52.5 | 2.00 | | 1 |
| Thibie, FR | 10.8 | 605 | MC | Loam | 1.36 | ND | Con | Winter wheat | Radish/winter cereal | NL | 123 | 17 | 23.5 | 50.2 | 51.8 | 1.60 | | 1 |
| Rio Grande do Sul, BR | 19.4 | 1440 | MW | Sandy clay loam | ND | ND | Con | corn | Oat/cowpea | M | 0.0 | 15 | 30 | 44.6 | 50.2 | 5.60 | | 2 |
| Rio Grande do Sul, BR | 19.4 | 1440 | MW | Sandy clay loam | ND | ND | NT | corn | Oat/cowpea | M | 0.0 | 15 | 30 | 49.2 | 56.6 | 7.40 | | 2 |
| Goias, BR | 22.5 | 1500 | MW | Clayey soil | 1.27 | 6.5 | NT | Rice/soya | Brachiaria | L | 111 | 6 | 30 | 21.5 | 22.3 | 0.84 | | 3 |
| Santa Maria, BR | 19.3 | 1769 | MW | Sandy loam | ND | 4.5 | Con /R | corn/grass/bare | Pigeon pea cover crops/cowpea | L | 120 | 10 | 20 | ND | ND | 6.16 | | 4 |
| Eldorado Sul, BR | 19.4 | 1440 | MW | Sandy clay loam | ND | 4.5 | Con /R | black oat/corn | Pigeon pea cover/cowpea | L | 144 | 17 | 20 | ND | ND | 7.08 | | 4 |
| Cruz Alta, BR | 19.2 | 1727 | MW | Clayey soil | ND | 4.5 | Con /NT | Wheat/soya bean/corn | Vetch/corn/radish | M | 73 | 19 | 20 | ND | ND | 7.47 | | 4 |
| Pisa, IT | 8.0 | 864 | MW | Loam soil | 1.45 | 8.2 | Con /NT | Wheat/soya bean/corn | Vetch/corn/radish | NL | 0-300 | 15 | 30 | 47.1 | 48.8 | 1.70 | | 5 |
| Pisa, IT | 8.0 | 864 | MW | Loam soil | 1.45 | 8.2 | Con /NT | Wheat/soya bean/corn | squarrosum clover | L | 0-300 | 15 | 30 | 47.1 | 48.6 | 1.50 | | 5 |
| Pisa, IT | 8.0 | 864 | MW | Loam soil | 1.45 | 8.2 | Con /NT | Wheat/soya bean/corn | squarrosum clover | L | 0-300 | 15 | 30 | 47.1 | 49.2 | 2.10 | | 5 |
| Wagga Wagga, AU | 15.8 | 561 | MW | ND | 1.8 | 5.0 | Con | Wheat/Oat | Cereal Rye | NL | 20 | 1 | 10 | 0.14 | 0.16 | 0.02 | | 6 |
| Wagga Wagga, AU | 15.8 | 561 | MW | ND | 1.8 | 5.1 | Con | Wheat/Oat | Wheat | NL | 20 | 1 | 10 | 0.14 | 0.17 | 0.03 | | 6 |
| Wagga Wagga, AU | 15.8 | 561 | MW | ND | 1.8 | 5.0 | Con | Wheat/Oat | Saia oat | NL | 20 | 1 | 10 | 0.14 | 0.17 | 0.03 | | 6 |
| Wagga Wagga, AU | 15.8 | 561 | MW | ND | 1.8 | 5.1 | Con | Wheat/Oat | Vetch | L | 20 | 1 | 10 | 0.14 | 0.17 | 0.03 | | 6 |
| Wagga Wagga, AU | 15.8 | 561 | MW | ND | 1.8 | 5.0 | Con | Wheat/Oat | Field peas | L | 20 | 1 | 10 | 0.14 | 0.17 | 0.03 | | 6 |
| Wagga Wagga, AU | 15.8 | 561 | MW | ND | 1.8 | 5.0 | Con | Wheat/Oat | Mustard | NL | 20 | 1 | 10 | 0.14 | 0.16 | 0.02 | | 6 |
| Wagga Wagga, AU | 15.8 | 561 | MW | ND | 1.8 | 5.1 | Con | Wheat/Oat | Mixture | M | 20 | 1 | 10 | 0.14 | 0.16 | 0.02 | | 6 |
| Florida, USA | 19.7 | 1380 | MW | Loam soil | 1.43 | 8.0 | ND | Bare soil | Sunn hemp | L | 55 | 1 | 20 | 61.59 | 65.52 | 3.93 | | 7 |
| Florida, USA | 19.7 | 1380 | MW | Loam soil | 1.43 | 8.0 | ND | Bare soil | Velvet bean | L | 55 | 1 | 20 | 61.59 | 64.21 | 2.62 | | 7 |
| Florida, USA | 19.7 | 1380 | MW | Loam soil | 1.43 | 8.0 | ND | Bare soil | Cowpea | L | 55 | 1 | 20 | 61.59 | 62.90 | 1.31 | | 7 |
| Florida, USA | 19.7 | 1380 | MW | Loam soil | 1.43 | 8.0 | ND | Bare soil | Sorghum Sudan-grass | NL | 55 | 1 | 20 | 61.59 | 65.52 | 3.93 | | 7 |
| Florida, USA | 19.7 | 1380 | MW | Loam soil | 1.43 | 8.0 | ND | Bare soil | Sunn hemp | L | 55 | 1 | 20 | 62.90 | 66.83 | 3.93 | | 7 |
| Florida, USA | 19.7 | 1380 | MW | Loam soil | 1.43 | 8.0 | ND | Bare soil | Velvet bean | L | 55 | 1 | 20 | 62.90 | 65.52 | 2.62 | | 7 |
| Florida, USA | 19.7 | 1380 | MW | Loam soil | 1.43 | 8.0 | ND | Bare soil | Cowpea | L | 55 | 1 | 20 | 62.90 | 64.21 | 1.31 | | 7 |
| Florida, USA | 19.7 | 1380 | MW | Loam soil | 1.43 | 8.0 | ND | Bare soil | Sorghum Sudan-grass | NL | 55 | 1 | 20 | 62.90 | 66.83 | 3.93 | | 7 |
| Fort Valley, USA | 17.7 | 1187 | MW | Sandy loam | 1.39 | 6.5 | NT | Tomato/egg plant | Hairy vetch | L | 0 | 6 | 20 | 29.09 | 32.63 | 3.54 | | 8 |
| Fort Valley, USA | 17.7 | 1187 | MW | Sandy loam | 1.39 | 6.5 | NT | Tomato/egg plant | Hairy vetch | L | 90 | 6 | 20 | 29.22 | 31.45 | 2.23 | | 8 |
| Fort Valley, USA | 17.7 | 1187 | MW | Sandy loam | 1.39 | 6.5 | NT | Tomato/egg plant | Hairy vetch | L | 180 | 6 | 20 | 29.48 | 30.79 | 1.31 | | 8 |
| Fort Valley, USA | 17.7 | 1187 | MW | Sandy loam | 1.39 | 6.5 | CH | tomato/egg plant | Hairy vetch | L | 0 | 6 | 20 | 29.09 | 28.96 | -0.13 | | 8 |
| Fort Valley, USA | 17.7 | 1187 | MW | Sandy loam | 1.39 | 6.5 | CH | Tomato/egg plant | Hairy vetch | L | 90 | 6 | 20 | 28.43 | 28.57 | 0.13 | | 8 |
| Fort Valley, USA | 17.7 | 1187 | MW | Sandy loam | 1.39 | 6.5 | CH | tomato/egg plant | Hairy vetch | L | 180 | 6 | 20 | 30.66 | 31.06 | 0.39 | | 8 |
| Fort Valley, USA | 17.7 | 1187 | MW | Sandy loam | 1.39 | 6.5 | Con | Tomato/egg plant | Hairy vetch | L | 0 | 6 | 20 | 27.26 | 28.04 | 0.79 | | 8 |
| Fort Valley, USA | 17.7 | 1187 | MW | Sandy loam | 1.39 | 6.5 | Con | Tomato/egg plant | Hairy vetch | L | 90 | 6 | 20 | 26.08 | 26.47 | 0.39 | | 8 |
| Fort Valley, USA | 17.7 | 1187 | MW | Sandy loam | 1.39 | 6.5 | Con | Tomato/egg plant | Hairy vetch | L | 180 | 6 | 20 | 26.08 | 27.65 | 1.57 | | 8 |
| Fort Valley, USA | 17.7 | 1187 | MW | Sandy loam | 1.39 | 6.5 | NT/CH/ Con | Tomato/egg plant | Rye | NL | 0 | 6 | 20 | 21.62 | 24.11 | 2.49 | | 8 |
| Fort Valley, USA | 17.7 | 1187 | MW | Sandy loam | 1.39 | 6.5 | NT/CH/ Con | Tomato/egg plant | Hairy vetch | L | 90 | 6 | 20 | 21.62 | 23.46 | 1.83 | | 8 |
| Fort Valley, USA | 17.7 | 1187 | MW | Sandy loam | 1.39 | 6.5 | NT/CH/ Con | Tomato/egg plant | Crimson clover | L | 180 | 6 | 20 | 21.62 | 23.32 | 1.70 | | 8 |
| Tifton, USA | 18.6 | 1192 | MW | Loamy sand | 1.55-1.78 | ND | RT | Corn | Sunn hemp/crimson clover | L | 58 | 3 | 2.5 | ND | ND | 1.10 | | 9 |
| Michoaca´n, MEX | 14.0 | 1100 | MW | ND | 0.60 | 6.0 | Con | Corn | Vetch | L | 0 | 3 | 25 | 7.30 | 6.90 | -0.40 | | 10 |
| Michoaca´n, MEX | 14.0 | 1100 | MW | ND | 0.60 | 6.0 | Con | Corn | Oat | NL | 0 | 3 | 25 | 7.30 | 7.30 | 0.00 | | 10 |
| Michoaca´n, MEX | 14.0 | 1100 | MW | ND | 0.60 | 6.0 | NT | Corn | Vetch | L | 0 | 3 | 25 | 7.30 | 7.50 | 0.20 | | 10 |
| Michoaca´n, MEX | 14.0 | 1100 | MW | ND | 0.60 | 6.0 | NT | Corn | Oat | NL | 0 | 3 | 25 | 7.30 | 7.50 | 0.20 | | 10 |

MAAT - mean annual air temperature (^o^C) and MAP - mean annual precipitation. C= primary crop; CC= cover crops. ^a^Different methods were used to measure soil pH using pH probe/ meter in deionized water or 0.01 M CaCl_2_ in 1:1 and 1:2, or 1:5 (v: v) soils: solution ratios. ¶= SOC at the top 30 cm was calculated using the depth distributions method (Jobbagy & Jackson, 2001). ND= no data available. *= differences in SOC between the control and cover crop treatments. Added N fertilizer is in kg N ha^-1^. MW= moist warm; MC= moist cool climate zone. L= legume; NL= non-legume and M= mixed. Con= conventional; NT= no-till and R= reduced tillage; CH= chisel ploughing. FR= France; BR= Brazil; USA= United Sates of America; MEX= Mexico; IT= Italy; AU= Australia. Ref: 1= Constantin et al. (2010); 2= Bayer et al. (2000); 3= Metay et al. (2007); 4= Amado et al. (2006); 5= Mazzoncini et al. (2011); 6= Zhou et al. (2010); 7= Wang et al. (2012); 8= Sainju et al. (2002); 9= Hubbard et al. (2013); 10= Astier et al. (2006).
