# Supplementary material for: A critical review of the impacts of cover crops on nitrogen leaching, net greenhouse gas balance and crop productivity
Source: Glob Chang Biol. 2019 May 13;25(8):2530–43. doi: 10.1111/gcb.14644 (PMC6851768; doi:10.1111/gcb.14644)
Supplement: Supplementary file 5 [file GCB-25-2530-s005.docx]

Table 5: Published studies on the impacts of cover crops, climate and soil properties on direct N_2_O emissions from soils.

| Location  (country/state) | MAAT  (^o^C) | | MAP  (mm) | Soil texture | BD  (g cm^-3^) | pH^a^ | Tillage system | Primary crop (C ) | Cover crops (CC) | Type of CC | Added N (kg ha^-1^) | Duration  (year) | | *Direct N_2_O emissions under C (kg ha^-1^) | Direct N_2_O emissions under (CC) (kg ha^-1^) | ∆ direct N_2_O emissions (kg N ha^-1^)** |  | Ref. | |
| --- | --- | --- | --- | --- | --- | --- | --- | --- | --- | --- | --- | --- | --- | --- | --- | --- | --- | --- | --- |
| Minnesota, USA | | ND | ND | Silt loam | ND | 6.4 | Con | Corn/ soybean | Clover | L | 155.7 | | 2 | 1.00 | 1.95 | 0.95 | 1 | |  |
| Lethbridge, CA | | 5.9 | 380 | Clay loam | ND | ND | Con | Spring wheat | Oil seed radish | L | 45 | | 2 | 0.24 | 0.24 | 0.01 | 2 | |  |
| Lethbridge, CA | | 5.9 | 380 | Clay loam | ND | ND | Con | Spring wheat | Rye | NL | 45 | | 2 | 0.24 | 0.27 | 0.03 | 2 | |  |
| England, UK | | 10 | 730 | Sandy loam | 1.23 | 7.8 | Con | Corn | Ryegrass | NL | 250 | | 54d | 0.77 | 0.92 | 0.14 | 3 | |  |
| England, UK | | 10 | 730 | Sandy loam | 1.23 | 7.8 | Con | Corn | Wheat | NL | 200 | | 23d | 0.74 | 0.45 | -0.29 | 3 | |  |
| England, UK | | 10 | 730 | Sandy loam | 1.23 | 7.8 | Con | Corn | Ryegrass | NL | 0 | | 54d | 0.61 | 0.53 | -0.08 | 3 | |  |
| England, UK | | 10 | 730 | Sandy loam | 1.23 | 7.8 | Con | Corn | Wheat | NL | 0 | | 23d | 0.18 | 0.16 | -0.02 | 3 | |  |
| Foulum, DK | | 7.3 | 627 | Loamy sand | 1.4 | ND | DD | Spring barley | Fodder radish | NL | 100 | | 9m | 1.57 | 3.86 | 2.29 | 4 | |  |
| Foulum, DK | | 7.3 | 627 | Loamy sand | 1.4 | ND | R | Spring barley | Fodder radish | NL | 100 | | 9m | 1.60 | 2.17 | 0.57 | 4 | |  |
| Foulum, DK | | 7.3 | 627 | Loamy sand | 1.4 | ND | NT | Spring barley | Fodder radish | NL | 100 | | 9m | 1.96 | 3.03 | 1.07 | 4 | |  |
| Iowa, USA | | ND | 763 | Silty clay loam/Clarion loam | 1.35 | 6.8 | NT | Corn | Ryegrass | NL | 210 | | 5 | 7.80 | 7.22 | -0.58 | 5 | |  |
| Iowa, USA | | ND | 757 | Silty clay loam/Clarion loam | 1.35 | 6.8 | NT | Soybean | Ryegrass | NL | 0 | | 5 | 4.42 | 3.66 | -0.76 | 5 | |  |
| Iowa, USA | | ND | ND | Silty clay loam/Clarion loam | 1.36 | 6.7 | NT | Corn/soybean | Ryegrass | NL | 202 | | 2 | 7.37 | 7.92 | 0.55 | 6 | |  |
| Iowa, USA | | ND | ND | Silty clay loam/Clarion loam | 1.36 | 6.7 | NT | Soybean/corn | Ryegrass | NL | 202 | | 2 | 6.75 | 8.84 | 2.09 | 6 | |  |
| Iowa, USA | | 8.7 | 835 | Fine-loamy soil | ND | 6 | NT | Corn/soybean | Rye /oat | M | 336 | | 1 | 3.68 | 2.32 | -1.36 | 7 | |  |
| Iowa, USA | | 8.7 | 835 | Fine-loamy soil | ND | 6 | NT | Corn/soybean | Rye /oat | M | 224 | | 1 | 1.50 | 1.32 | -0.18 | 7 | |  |
| Iowa, USA | | 8.7 | 835 | Fine-loamy soil | ND | 6 | ND | Corn/soybean | Rye /oat | M | 112 | | 1 | 0.91 | 1.06 | 0.15 | 7 | |  |
| Jinju, SK | | ND | ND | Clay loam | ND | 6.5 | ND | Bare soil | Barley | NL | 0 | | 1 | 3.60 | 3.40 | -0.20 | 8 | |  |
| Jinju, SK | | ND | ND | Clay loam | ND | 6.5 | ND | Bare soil | Vetch | L | 0 | | 1 | 3.60 | 6.30 | 2.70 | 8 | |  |
| Jinju, SK | | ND | ND | Clay loam | ND | 6.5 | ND | Bare soil | Barley/vetch | M | 0 | | 1 | 3.60 | 6.00 | 2.40 | 8 | |  |
| New york, USA | | 9 | 819 | ND | ND | ND | ND | Corn | Clover | L | 176.2 | | 1 | 4.00 | 3.60 | -0.40 | 9 | |  |
| New york, USA | | 9 | 819 | ND | ND | ND | ND | Corn | Clover | L | 176.2 | | 1 | 1.80 | 3.0 | 1.20 | 9 | |  |
| Foulum, DK | | 7.3 | 627 | Loamy sand | 1.35 | 6.5 | Con | spring barley/grass-clover/potato/w wheat | Grass/clover | M | 277 | | 1 | 2.6 | 3.00 | 0.40 | 10 | |  |
| Foulum, DK | | 7.3 | 627 | Loamy sand | ND | ND | Con | spring barley/grass-clover/potato/w wheat | Grass/clover | M | 0 | | 1 | 3.20 | 3.7 | 0.50 | 10 | |  |
| Scotland, UK | | 5.6 | 730 | Sandy loam/loamy | ND | ND | Con | Oat | Rye/peas/barley/mustard and others | M | ND | | 53 d | 0.46 | 0.5 | 0.03 | 11 | |  |
| Scotland, UK | | 5.6 | 730 | Sandy loam/loamy | ND | 8.1 | Con | Oat | Rye/peas/barley/mustard and others | M | ND | | 19 d | 0.061 | 0.03 | -0.03 | 11 | |  |
| Turin, IT | | 11.9 | 734 | Silt loam | 1.35 | 6.5 | Con | Corn | Vetch | L | 130 | | 2 | 0.64 | 3.8 | 3.16 | 12 | |  |

MAAT - mean annual air temperature (^o^C) and MAP - mean annual precipitation. * N_2_O flux is cumulative when the duration < one year and annual when the duration is one year or more. **= differences in direct N_2_O emissions between the control and cover crop treatments. C = control; CC= cover crops (CC). ^a^Different methods were used to measure soil pH using pH probe/ meter in deionized water or 0.01 M CaCl_2_ in 1:1 and 1:2, or 1:5 (v: v) soils: solution ratios. ND= no data available; Con= conventional; R= reduced; NT= no-till; DD= direct drill. Added N fertilizer is in kg N ha^-1^. L= legume; NL= non-legume and M= mixed. USA= United States of America; CA= Canada; DK= Denmark; SK = South Korea; UK= United Kingdom; IT = Italy. Ref.:1= Turner et al. (2016); 2= Thomas et al. (2017); 3= Sarkodie-Addo et al. (2003); 4= Petersen et al. (2011); 5= Parkin et al. (2016); 6= Parkin et al. (2006); 7= Jarecki et al. (2009); 8= Hwang et al. (2017); 9= Han et al. (2017); 10= Brozyna et al. (2013); 11= Baggs et al. (2000); 12= Alluvione et al. (2010).

**References**

Alluvione, F., Bertora, C., Zavattaro, L. & Grignani, C. (2010). Nitrous oxide and carbon

dioxide emissions following green manure and compost fertilization in corn. *Soil Science Society of America Journal, 74(2)*, 384-395.

Amado, T.J.C., Bayer, C., Conceição, P.C., Spagnollo, E., Campos, B.C. & Veiga, M. (2006).

Potential of carbon sequestration in no-till soils with intensive use and cover crops in

the southern Brazil. *Journal of Environmental Quality 35,* 1599-1607.

Amossé, C., Jeuffroy, M.H., Mary, B. & David, C. (2014). Contribution of relay

intercropping with legume cover crops on nitrogen dynamics in organic grain systems. *Nutrient Cycling in Agroecosystems, 98*, 1-14.

Aronsson, H., Ringselle, B., Andersson, L. & Bergkvist, G. (2015). Combining mechanical

control of couch grass (*Elymus repens* L.) with reduced tillage in early autumn and cover crops to decrease nitrogen and phosphorus leaching. *Nutrient Cycling in Agroecosystems, 102,* 383-396.

Aronsson, H., Stenberg, M. & Ulén, B. (2011). Leaching of N, P and glyphosate from two

soils after herbicide treatment and incorporation of a ryegrass catch crop. *Soil Use and*

*Management, 27,* 54-68.

Askegaard, M. & Eriksen, J. (2008). Residual effect and leaching of N and K in cropping

systems with clover and ryegrass catch crops on coarse sand. [*Agriculture, Ecosystems and Environ*](http://www.sciencedirect.com/science/journal/01678809)*ment, 123,* 99-108.

Askegaard, M., Olesen, J.E., Rasmussen, I.A. & Kristensen, K. (2011). Nitrate leaching from

organic arable crop rotations is mostly determined by autumn field management.

[*Agriculture, Ecosystems and Environ*](http://www.sciencedirect.com/science/journal/01678809)*ment, 142,* 149-160.

Askegaard, M., Olesen, J.E., Rasmussen, I.A. & Kristensen, K. (2005). Nitrate leaching from

organic arable crop rotations: effects of location, manure and catch crop. *Soil Use and*

*Management, 21,*181-188.

Astier, M., Maass, J.M., Etchevers-Barra, J.D., Peña, J.J. & González, F.D.L. (2006). Short-

term green manure and tillage management effects on maize yield and soil quality in an Andisol. *Soil and Tillage Research, 88,* 153-159.

Baggs, E.M., Watson, C.A. & Rees, R.M. (2000). The fate of nitrogen from incorporated

cover crop and green manure residues. *Nutrient Cycling in Agroecosystems, 56(2),*

153-163.

Bai, J.S., Cao, W.D., Xiong, J., Zeng, N.H., Gao, S.J. & Shimizu, K. (2015). Integrated

application of February Orchid (*Orychophragmus violaceus*) as green manure with chemical fertilizer for improving grain yield and reducing nitrogen losses in spring maize system in northern China. *Journal of Integrative Agriculture, 14 (12),* 2490-2499.

Bayer, C., Mielniczuk, J., Amado, T.J.C., Martin-Neto, L. & Fernandes, S.V. (2000).

**Organic matter storage in a sandy clay loam Acrisol affected by tillage and cropping systems in southern Brazil.** *Soil and Tillage Research, 54,*101-109.

Benoit, M., Garnier, J., Anglade, J. & Billen, G. (2014). Nitrate leaching from organic and

conventional arable crop farms in the Seine Basin (France). *Nutrient Cycling in Agroecosystems,* *100,* 285-299.

Bergström, L., & Jokela, W. E. (2001). Ryegrass cover crop effects on nitrate leaching in

spring barley fertilized with ^15^NH_4_ ^15^NO_3_. *Journal of Environmental Quality, 30,* 1659-1667.

Berntsen, J., Olesen, J.E., Petersen, B.M. & Hansen, E.M. (2006). Algorithms for sensor-

based redistribution of nitrogen fertilizer in winter wheat. *Precision Agriculture, 7,* 65-83.

Brozyna, M.A., Petersen, S.O., Chirinda, N. & Olesen, J.E. (2013). Effects of grass-clover

management and cover crops on nitrogen cycling and nitrous oxide emissions in a stockless organic crop rotation. *Agriculture, Ecosystems and Environment,* *181,* 115-126.

Constantin, J., Mary, B., Laurent, F., Aubrion, G., Fontaine, A., Kerveillant, P. & Beaudoin,

N. (2010). Effects of catch crops, no till and reduced nitrogen fertilization on nitrogen leaching and balance in three long-term experiments. *Agriculture, Ecosystems and Environment, 135,* 268-278.

Coombs, C., [Lauzon, J.D.,](https://www.sciencedirect.com/science/article/pii/S0378429016306074#!) [Deen, B.](https://www.sciencedirect.com/science/article/pii/S0378429016306074#!) &Van Eerd, L.L. (2017). Legume cover crop

management on nitrogen dynamics and yield in grain corn systems. *Field Crops Research, 20,* 75-85.

Doltra, J. & Olesen, J. (2013). The role of catch crop in the ecological intensification of

spring cereals in organic farming under Nordic climate. *European Journal of Agronomy, 44,* 98-108.

Engstrom, L., Stenberg, M., Aronsson, H. & Linden, B. (2011). Reducing nitrate leaching

after winter oilseed rape and peas in mild and cold winters. *Agronomy for Sustainable Development, 31,* 337-347.

Francis, G.S. (1995). Management practices for minimizing nitrate leaching after ploughing

temporary leguminous pastures in Canterbury, New Zealand. *Journal of Contaminant Hydrology, 20,* 313-327.

Fraser, P.M., Curtin, D., Harrison-kirk, T., Meenken, E.D., Beare, M.H., Tabley, F.,

Gillespie, R.N. & Francis, G.S. (2013). Winter nitrate leaching under different tillage

and winter cover crop management practices. *Soil Science Society of America Journal, 77,* 1391-1401.

Guo, R., Li, X. L., Christie, P., Chen, Q., Jiang, R. F. & Zhang, F.S. (2008). Influence of root

zone nitrogen management and a summer catch crop on cucumber yield and soil mineral nitrogen dynamics in intensive production systems. *Plant and Soil, 313 (1-2),* 55-70.

Han, Z., Walter, M.T. & Drinkwater, L.E. (2017). Impact of cover cropping and landscape

positions on nitrous oxide emissions in north-eastern US agroecosystems. *Agriculture, Ecosystems and Environment, 245*, 24-34.

Hansen, E.M. & Djurhuus, J. (1997). Nitrate leaching as influenced by soil tillage and catch

crop. *Soil and Tillage Research, 41*, 203-219.

Hooker, K.V., Coxon, C.E. Hackett, R., Kirwan, L.E., O’Keeffe, E. & Richards, K.G. (2008).

Evaluation of cover crop and reduced cultivation for reducing nitrate leaching in Ireland. *Journal of Environmental Quality, 37,* 138-145.

Hubbard, R.K., Strickland, T.C. & Phatak, S. (2013). Effects of cover crop systems on soil

physical properties and carbon/nitrogen relationships in the coastal plain of south-eastern USA. *Soil and Tillage Research, 126,* 276-283.

Hu, X., Su, F., Ju, X., Gao, B., Oenema, O., Christie, P., Huang, B., Jiang, R. & Zhang, F.

(2013). Greenhouse gas emissions from a wheat-maize double cropping system with different nitrogen fertilization regimes. *Environmental Pollution, 176,* 198-207.

Hwang, H.Y., Kim, G.W., Kim, S.Y., Haque, Md. M., Khan, M.I. & Kim, P.J. (2017). Effect

of cover cropping on the net global warming potential of rice paddy soil. *Geoderma, 292,* 49-58.

Jarecki, M.K., Parkin, T.B., Chan, A.S., Kaspar, T.C., Moorman, T.B., Singer, J.W., Kerr,

B.J., Hatfield, J.L. & Jones, R. (2009). Cover crop effects on nitrous oxide emission from a manure-treated Mollisol. *Agriculture, Ecosystems and Environment, 134(1-2),* 29-35.

Jobbagy, E.G., Jackson, R.B. (2001). The distribution of soil nutrients with depth: global

patterns and the imprint of plants. *Biogeochemistry*, *53*, 51-77.

Känkänen, H. & Eriksson, C. (2007). Effects of undersown crops on soil mineral N and grain

yield of spring barley. *European Journal of Agronomy, 27,* 25-34.

Kaspar, T.C., Jaynes, D.B., Parkin, T.B., Moorman, T.B. & Singer, J.W. (2012).

Effectiveness of oat and rye cover crops in reducing nitrate losses in drainage water. *Agricultural Water Management, 110,* 25-33.

Kramberger, B., Gselman, A., Janzekovic, M., Kaligaric, M. & Bracko, B. (2009). Effects of

cover crops on soil mineral nitrogen and on the yield and nitrogen content of maize. *European Journal of Agronomy, 31,* 103-109.

Lemola, R. & Turtola, E. (2000). Under-sowing Italian ryegrass diminishes N leaching from

spring barley. *Agricultural and Food Science in Finland*, *9,* 201-215.

Lewan, E. (1994). Effects of a catch crop on leaching of nitrogen from a sandy loam:

Simulations and measurements. *Plant and Soil 166,*137-152.

Liang, X., Zhang, H., He, M., Yuan, J., Xu, L. & Tian, G. (2016). No-tillage effects on grain

yield, N use efficiency, and nutrient runoff losses in paddy fields. *Environment Science and Pollution Research, 23,* 21451-21459.

Li, S. L., Li, D. R. & Hu, C. (2012). Impact of reducing chemical fertilizer combined with

Chinese milk vetch on growth and yield of double cropping rice. *Soil Fertilizer Sciences in China, 1,* 69-73. (in Chinese with English abstract).

Liu, C. Z., Liu, X. F., Ben-Yin, L. I., et al. (2012). Effects of planting Chinese milk vetch on

rice yield, soil aggregation and distributions of its carbon and total nitrogen. *Acta Agriculturae Boreali-Sinica, 27(6),* 224-228. (in Chinese with English abstract).

Lu, X., Gilliam, F.S., Yu, G., Li, L., Mao, Q., Chen, H. & Mo, J. (2013). Long-term nitrogen

addition decreases carbon leaching in a nitrogen-rich forest ecosystem. *Biogeosciences, 10,* 3931-3941.

Malecka, I. & Blecharczyk, A. (2008). Effect of tillage systems, mulches and nitrogen

fertilization on spring barley (Hordeum vulgare). *Agronomy Research, 6,* 517-529.

Mao, et al. (2015). Preliminary Study of Catch Crop Growing in Greenhouse in Yellow River

Irrigation Area of Ningxia, Ningxia. *Journal of Agricultural Sciences and Technology, 56(09),* 1-4. (in Chinese with English abstract).

Mazzoncini, M., Sapkota, T.B., Barberi, P., et al. (2011). Long-term effect of tillage, nitrogen

fertilization and cover crops on soil organic carbon and total nitrogen content. *Soil and Tillage Research, 114,* 165-174.

McLenaghen, R.D., Cameron, K.C., Lampkin, N.H., Daly, M.L. & Deo, B. (1996). Nitrate

leaching from ploughed pasture and the effectiveness of winter catch crops in reducing leaching losses. N.Z.J. *Agricultural Research, 39(3),* 413-420.

Metay, A., Moreira, J.A.A., Bernoux, M., Boyer, T., Douzet, J-M., Feigl, B., Feller, C.,

Maraux, F., Oliver, R. & Scope, E. (2007). Storage and forms of organic carbon in a no-tillage under cover crops system on clayey oxisol in dryland rice production*. Soil and Tillage Research, 94,*122-132.

Min, J., Zhao, X., Shi, W., Xing, G. & Zhu, Z. (2011). Nitrogen Balance and Loss in a

Greenhouse Vegetable System in Southeastern China. *Pedosphere, 21 (4),* 464-472.

Parkin, T.B., Kaspar, T.C. & Singer, J.W. (2006). Cover crop effects on the fate of N

following soil application of swine manure. *Plant and Soil, 289(1-2),* 141-152.

Peng et al. (2015). Effects of Catch Crops on Reducing Soil Nitrate Accumulation and

Cucumber Growth in Greenhouse Vegetable Production System. *Scientia Agricultura Sinica, 48(9),* 1774-1784. (in Chinese with English abstract).

Petersen, S.O., Mutegi, J.K., Hansen, E.M. & L.J. Munkholm. (2011). Tillage effects on N_2_O

emissions as influenced by a winter cover crop. *Soil Biology and Biochemistry, 43(7),* 1509-1517.

Qiao, J., Yan, T. M., Xue, F., et al. (2011). Reduction of nitrogen fertilizer application under

different crop rotation systems in paddy fields of Taihu Area. *Chinese Journal of Eco-agriculture,19(1),* 24-31. (in Chinese with English abstract).

Ren et al. (2006). Soil nitrogen up take by sweet corn to reduce nitrogen leaching in the

vegetable field. *Transactions of the CSAE 22(9),* 245- 249. (in Chinese with English abstract).

Richards, I. R., Wallace, P. A. & Turner, I. D. S. (1996). A comparison of six cover crop

types in terms of nitrogen uptake and effect on response to nitrogen by a subsequent spring barley crop. *Journal of Agricultural Sciences, 127,* 441-449.

Rinnofner, T., Friedel, J.K., de Kruijff, R., Pietsch, G. & Freyer, B. (2008). Effect of catch

crops on N dynamics and following crops in organic farming. *Agronomy for Sustainable Development, 28,* 551-558.

Ritter, W.F., Scarborough, R.W. & Chirnside, A.E.M. (1998). Winter cover crops as best

management practice for reducing nitrate leaching. *Journal of Contaminant Hydrology, 34,* 1-15.

Sainju, U.M., Singh, B.P. & Whitehead, W.F. (2002). Long-term effects of tillage, cover

crops, and nitrogen fertilization on organic carbon and nitrogen concentrations in sandy loam soils in Georgia, USA. *Soil and Tillage Research, 63,* 167-179.

Salmerón, M., Cavero, J., Quilez, D. & Isla, R. (2010). Winter cover crops affect

monoculture maize yield and nitrogen leaching under irrigated Mediterranean conditions. *Agronomy Journal, 102,* 1700-1709.

Sarkodie-Addo, J., Lee, H.C. & Baggs, E.M. (2003). Nitrous oxide emissions after

application of inorganic fertilizer and incorporation of green manure residues. *Soil Use and Management, 19*, 331-339.

Stenberg, M., Aronsson, H., Lindén, B., Rydberg, T. & Gustafson, A. (1999). Soil mineral

nitrogen and nitrate leaching losses in soil tillage systems combined with a catch crop. *Soil and Tillage Research, 50,* 115-125.

Tang, H. M., Xiao, X. P., Tang, W. G., et al. (2010). Effects of different winter cover crops

on paddy soil nutrients and growth of rice in southern China. *Acta Agriculturae Universitatis Jiangxiensis, 32(1),* 9-8. (in Chinese with English abstract).

Teixeira, E.I., Johnstone, P., Chakwizira, E., de Ruiter, J., Malcolm, B., Shaw, N.,

Zyskowski, R., Khaembah, E., Sharp, J., Meenken, E., Fraser, P., Thomas, S., Brown, H. & Curtin, D. (2016). Sources of variability in the effectiveness of winter cover crops for mitigating N leaching. *Agriculture Ecosystems, and Environment, 220,* 226-235.

Thomas, B.W., Goyer, C., Chantigny, M.H. & Charles, A. (2017). Non-legume cover crops

can increase non-growing season nitrous oxide emissions. *Soil Science Society of America Journal, 81,* 189-199.

Thomsen, I.K. (2005). Nitrate leaching under spring barley is influenced by the presence of a

ryegrass catch crop: results from a lysimeter experiment. *Agriculture Ecosystems, and Environment,* *111,* 21-29.

Thomsen, I.K. & Hansen, E.M. (2014). Cover crop growth and impact on N leaching as

affected by pre- and postharvest sowing and time of incorporation. *Soil Use and Management, 30,* 48-57.

Torstensson, G. & Aronsson, H. (2000). Nitrogen leaching and crop availability in manured

catch crop systems in Sweden. *Nutrient Cycling in Agroecosystems, 56,* 139-152.

Torstensson, G., Aronsson, H. & Bergström, L. (2006). Nutrient use efficiencies and

leaching of organic and conventional cropping systems in Sweden. *Agronomy Journal, 98,* 603-615.

Turner, P.A., Baker, J.M., Griffis, T.J. & Venterea, R.T. (2016). Impact of Kura Clover living

mulch on nitrous oxide emissions in a corn-soybean system. *Journal of Environmental Quality, 45*, 1782-1787.

Wang, D., Li, H., Wei, Z., et al. (2006). Effect of earthworms on the phytoremediation of

zinc-polluted soil by ryegrass and Indian mustard*. Biology and Fertility of Soils, 43(1),* 120-123.

Wang, Q., Li, Y., Klassen, W. & Alva, A. (2012). High retention of N P nutrients, soil

organic carbon, and fine particles by cover crops under tropical climate. *Agronomy for Sustainable Development, 32,* 781-790.

Welch, R.Y., Behnke, G.D., Davis, A.S., Masiunas, J. & Villamil, M.B. (2016). Using cover

crops in headlands of organic grain farms: Effects on soil properties, weeds and crop yields. *Agriculture Ecosystems, and Environment, 216,* 322-332.

Wyland, L.J., Jackson, L.E., Chaney, W.E., Klonsky, K., Koike, S.T. & Kimple, B. (1996).

Winter cover crops in a vegetable cropping system: Impact on nitrate leaching, soil water, crop yield, pests and management costs. *Agriculture Ecosystems, and Environment, 59,* 1-17.

Xi et al. (2011). Effect of Sweet Maize as Catch Crop on Soil Environment and Following

Vegetable in Greenhouse of Northern China. *Journal of Agro-Environment Science 30(1),* 113-119. (in Chinese with English abstract).

Zhang, G. X. & Xie, W. (2006). Effects of straw mulching on Maize Yield and soil fertility.

Mod. *Journal of Agricultural Sciences and Technology, 5,* 58-59. (in Chinese with English abstract).

Zhang, S., Lövdahl, L., Grip, H., Tong, Y., Yang, X. & Wang, Q. (2009). Effects of mulching

and catch cropping on soil temperature, soil moisture and wheat yield on the Loess Plateau of China. *Soil and Tillage Research, 102 (1),* 78-86.

Zhou, X., Wu, H., Xu, Z. & Chen, C. (2010). Winter cover crops increase soil carbon and

nitrogen cycling processes and microbial functional diversity. ^19^th World Congress of Soil Science, Soil Solutions for a Changing World 47 1-6 August 2010, Brisbane, Australia. Published on DVD.

Zhu, B., Yi, L., Xu, H., Guo, L., Hu, Y., Zeng, Z., Chen, F. & Liu, Z. (2016). Non-

leguminous winter cover crop and nitrogen rate in relation to double rice grain yield and nitrogen uptake in Dongting Lake Plain, Hunan Province, China. *Journal of Integrative Agriculture, 15(11),* 2507-2514.

Zhu, G. P., Zhang, H. Q., Zeng-Qi, W. U., et al. (2011). Effects of Astragalus sinicus

Ploughed at Different Growth Stages on Its Nitrogen Content and Rice Yield. *Acta Agriculturae Jiangxi, 23 (2),* 122-124. (in Chinese with English abstract).
